# Intermediates and shunt products of massiliachelin biosynthesis in *Massilia* sp. NR 4-1

**DOI:** 10.3762/bjoc.19.69

**Published:** 2023-06-23

**Authors:** Till Steinmetz, Blaise Kimbadi Lombe, Markus Nett

**Affiliations:** 1 Department of Biochemical and Chemical Engineering, Laboratory of Technical Biology, TU Dortmund University, Emil-Figge-Strasse 66, 44227 Dortmund, Germany

**Keywords:** *Massilia*, massiliachelin, siderophore, structure elucidation

## Abstract

Siderophores are small molecules secreted by microorganisms in order to scavenge iron from the environment. An example is the thiazoline-containing natural product massiliachelin, which is produced by *Massilia* sp. NR 4-1 under iron-deficient conditions. Based on experimental evidence and genome analysis, it was suspected that this bacterium synthesizes further iron-chelating molecules. After a thorough inspection of its metabolic profile, six previously overlooked compounds were isolated that were active in the chrome azurol S (CAS) assay. Mass spectrometric measurements and nuclear magnetic resonance spectroscopic analyses identified these compounds as possible biosynthetic intermediates or shunt products of massiliachelin. Their bioactivity was tested against one Gram-positive and three Gram-negative bacteria.

## Introduction

Iron is crucial for many important biological processes, such as photosynthesis, respiration or nitrogen fixation, in which iron-containing proteins are engaged in electron transfer reactions. In fact, the transition metal is perfectly suited for shifting electrons due to its ability to easily interconvert between a reduced ferrous (Fe^2+^) and an oxidized ferric state (Fe^3+^) [1]. To maintain iron homeostasis, all living organisms need to regulate the intake of this essential element from the environment. In bacteria, this is typically achieved through the use of siderophores [2], which are small molecules that are secreted under iron-limiting conditions to solubilize and chelate environmental Fe^3+^. Ligand groups, such as hydroxamate, phenolate, catecholate, carboxylate, or oxazoline/thiazoline residues, confer siderophores their high affinity for the binding of Fe^3+^ [3–5]. Following the coordination of the metal, the Fe^3+^-loaded siderophore complex is transported back into the cell through membrane receptors and transporters. Eventually, the bound metal is released through reductive or hydrolytic mechanisms [2].

**Figure 1:**
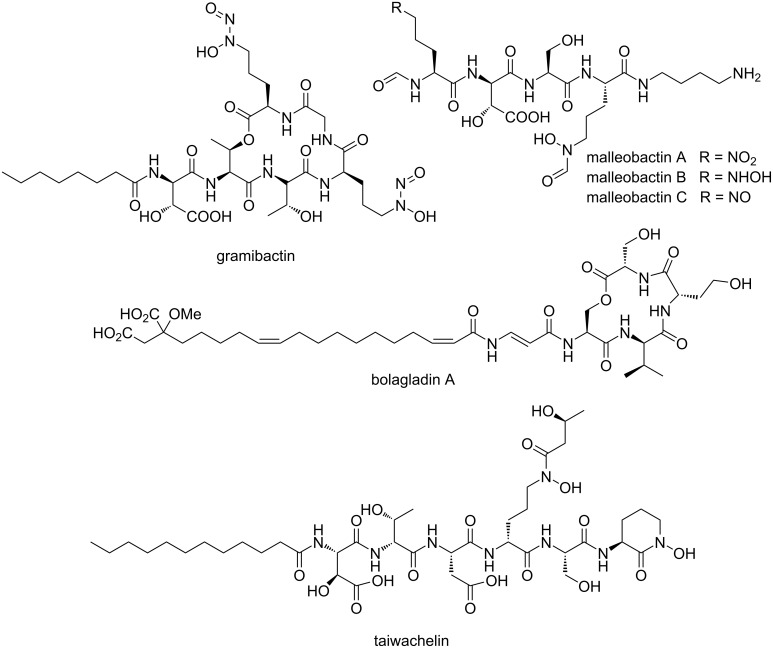
Selected siderophores from β-proteobacteria.

In the past years, β-proteobacteria have received increasing attention as producers of siderophores with interesting chemical features. For instance, *Burkholderia thailandensis* produces malleobactins (Figure 1), which possess nitro, nitroso, and azoxy groups [6]. Although the malleobactins are weaker iron chelators than related siderophores featuring hydroxamate groups [7], recent evidence suggests that their structural peculiarities might be of relevance for microbial communication processes [8]. Another unusual siderophore, gramibactin, is released by the rhizosphere bacterium *Paraburkholderia graminis* [9]. Gramibactin features an extremely rare diazeniumdiolate ligand with potent complexing properties [9–10]. Noteworthy is also bolagladin from *Burkholderia gladioli*, which possesses an unprecedented citrate-derived fatty acid moiety [11]. Furthermore, lipopeptide siderophores with photocleavable moieties, like taiwachelin, were reported from bacteria of the genera *Cupriavidus* and *Variovorax* [12–14].

The β-proteobacterial genus *Massilia* was recently identified as an untapped reservoir of novel natural products [15]. In particular, the strain *Massilia* sp. NR 4-1 was subject of several chemical investigations. In these studies, the bacterium was demonstrated to produce a diverse array of secondary metabolites, namely the pigment violacein [16], the cyclic guanidine alkaloid massinidine [17], and the siderophore massiliachelin [18]. An antiSMASH analysis [19] of the genome of *Massilia* sp. NR 4-1 revealed the presence of additional biosynthetic gene clusters, including another putative metallophore gene cluster (ACZ75_RS05545–ACZ75_RS06020). Although the architecture of the corresponding locus suggested an involvement in the production of a lipopeptide siderophore, the gene cluster could not be unequivocally associated with a known compound. The assumption that *Massilia* sp. NR 4-1 synthesizes iron-chelating molecules in addition to massiliachelin was further supported by laboratory experiments involving the chrome azurol S (CAS) assay [20]. For these reasons, *Massilia* sp. NR 4-1 appeared to be a promising candidate for the discovery of further siderophores. The renewed analysis led to the identification of six previously overlooked iron-chelating molecules, which are structurally and likely also biosynthetically linked to massiliachelin. However, these compounds are not related to the predicted lipopeptide siderophore. Herein, we describe their isolation, characterization and antibacterial activities, and we discuss their biosynthetic relationship to massiliachelin.

## Results and Discussion

**Figure 2:**
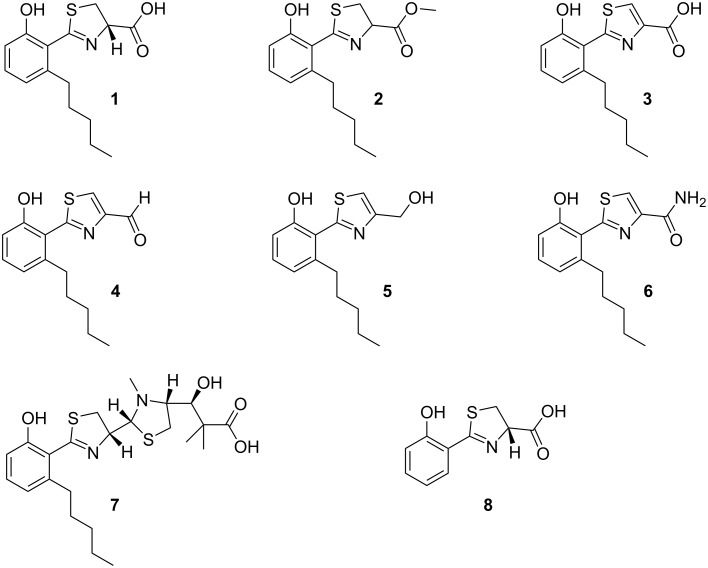
Chemical structures of compounds **1–6** isolated in this study and of the structurally related siderophores massiliachelin (**7**) and (*S*)-dihydroaeruginoic acid (**8**).

*Massilia* sp. NR 4-1 was cultivated in modified R2A medium under iron-replete conditions. As opposed to our former study [18], sodium pyruvate was not included in the medium, as this ingredient had been observed to enhance the production of violacein (Supporting Information File 1, Figure S1). In the absence of sodium pyruvate, the identification and isolation of minor metabolites from *Massilia* sp. NR 4-1 is facilitated, as already observed in the discovery of massinidine [17]. In the present study, several batch fermentations with a total culture volume of 12 L were carried out to secure sufficient material for structure elucidation. The metabolites secreted into the culture broth were recovered post-fermentation with the adsorber resin XAD-7. After the removal of the culture supernatant by filtration, the adsorbed compounds were eluted from the resin with methanol. The resulting extract was concentrated to dryness. For an initial separation of its components, the raw extract was resuspended in water and extracted three times with ethyl acetate. The organic phases were pooled. A testing in the CAS assay indicated the presence of iron-chelating molecules only in the organic phase but not in the aqueous phase. Therefore, the organic phase was concentrated under reduced pressure and the resulting residue was subjected to reversed-phase HPLC. Those fractions that showed a color change from blue to pink in the CAS assay were collected and purified in a second reversed-phase HPLC run. This led to the isolation of six compounds (**1–6**, Figure 2).

**Figure 3:**
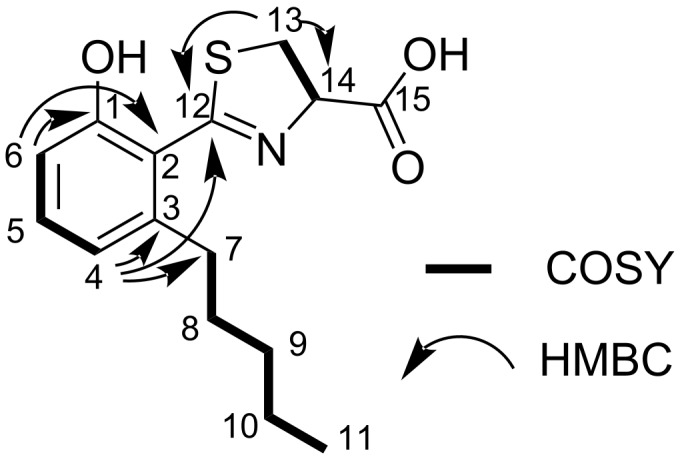
^1^H,^1^H-COSY and selected ^1^H,^13^C-HMBC correlations in **1**.

The NMR spectroscopic data of compound **1** (brownish oil, 1.7 mg) were found to be very similar to those of the previously reported massiliachelin [18], suggesting a structural relatedness. The empirical formula of **1** was assigned to be C_15_H_19_NO_3_S by high-resolution (HR) ESIMS (*m*/*z* 294.1180 [M + H]^+^; calcd. 294.1169 for C_15_H_20_NO_3_S), which indicated the presence of seven double bond equivalents (DBEs). Upon analyzing the ^13^C NMR spectrum, the DBEs were assigned to two ring structures, two carbon–heteroatom double bonds (δ_C_ 171.9, 167.1 ppm; Table 1) and three carbon–carbon double bonds. The latter involved carbon atoms resonating at δ_C_ 155.4, 142.3, 130.3, 119.9, 119.8, and 113.2 ppm. The signals in the ^1^H NMR spectrum of **1** could be distributed among three discrete spin systems according to proton–proton correlation spectroscopy (COSY; Figure 3).

**Table 1:** ^1^H and ^13^C NMR spectroscopic data for **1–6** in DMSO-*d*_6_.

No.	**1**			**2**			**3**	
					
	δ_H_, M (*J* in Hz)	δ_C_		δ_H_, M (*J* in Hz)	δ_C_		δ_H_, M (*J* in Hz)	δ_C_

1	–	155.4		–	155.3		–	155.8
2	–	119.8		–	119.8		–	119.1
3	–	142.3		–	142.2		–	143.4
4	6.69 dd (1.1, 7.8)	119.9		6.70 dd (1.1, 7.8)	119.9		6.79 dd (1.2, 7.7)	120.6
5	7.15 t (7.8)	130.3		7.15 t (7.8)	130.4		7.21 dd (7.7, 8.2)	130.5
6	6.70 dd (1.1, 7.8)	113.2		6.71 dd (1.1, 7.8)	113.2		6.83 dd (1.2, 8.2)	113.4
7	2.54 m 2.62 m	32.7		2.58 m	32.6		2.62 dd (3.8, 7.8)	33.2
8	1.49 m	31.0		1.49 m	30.9		1.38 m	30.2
9	1.25 m	31.2		1.25 m	31.2		1.17 m	31.0
10	1.25 m	21.9		1.29 m	21.9		1.17 m	21.7
11	0.85 t (7.0)	13.9		0.85 t (6.3)	13.9		0.77 t (7.1)	13.8
12	–	167.1		–	167.6		–	163.3
13	3.63 dd (8.1, 11.1) 3.69 dd (9.6, 11.1)	35.4		3.63 dd (8.3, 11.2) 3.72 dd (9.7, 11.2)	35.3		8.55 s	129.5
14	5.25 dd (8.1, 9.6)	77.6		5.33 dd (8.2, 9.7)	77.5		–	146.3
15	–	171.9		–	170.9		–	162.3
16				3.73 s	52.3			

No.	**4**			**5**			**6**	
					
	δ_H_, M (*J* in Hz)	δ_C_		δ_H_, M (*J* in Hz)	δ_C_		δ_H_, M (*J* in Hz)	δ_C_

1	–	155.7		–	156.5		–	155.7
2	–	118.8		–	119.9		–	119.3
3	–	143.5		–	143.5		–	143.5
4	6.81 dd (1.2, 7.8)	120.8		6.78 dd (1.2, 7.6)	121.0		6.78 dd (1.1, 7.9)	120.5
5	7.23 dd (7.8, 8.2)	130.8		7.19 dd (7.6, 8.2)	130.5		7.20 t (7.9)	130.5
6	6.84 dd (1.2, 8.2)	113.4		6.81 dd (1.2, 8.2)	114.1		6.81 dd (1.1, 7.9)	113.3
7	2.65 m	33.3		2.61 m	33.9		2.59 m	33.2
8	1.39 m	30.3		1.42 m	30.8		1.38 m	30.2
9	1.17 m	31.1		1.20 m	31.7		1.16 m	31.1
10	1.16 m	21.7		1.21 m	22.3		1.15 m	21.7
11	0.77 t (7.1)	13.8		0.81 t (6.9)	14.3		0.76 t (7.1)	13.8
12	–	164.4		–	163.5		–	163.1
13	8.84 s	132.6		7.53 s	115.8		8.36 s	125.3
14	–	153.7		–	157.0		–	149.5
15	9.98 s	184.9		4.63 s	60.2		–	162.4

The first spin system is part of a 2,3-substituted phenol moiety featuring proton signals at δ_H_ 7.15 (H-5), 6.70 (H-6) and 6.69 ppm (H-4; Table 1). Three aromatic carbon atoms could be assigned due to heteronuclear multiple bond correlation (HMBC) correlations from H-6 to 155.4 ppm (C-1) and 119.8 ppm (C-2) as well as from H-4 to 142.3 ppm (C-3) and 119.8 ppm (C-2). The upfield shifted resonance of H-6 suggested an electron-donating substituent at C-1 and the chemical shift of the latter (δ_C_ 155.4 ppm) supported the assignment of an hydroxy function in this position. An HMBC correlation from H-4 to the carbon atom at 32.7 ppm (C-7) allowed the linkage of the phenol moiety with an *n*-pentyl sidechain in *meta* position to the hydroxy group. The spin system of the latter includes proton resonances at δ_H_ 2.54 (H-7a), 2.62 (H-7b), 1.49 (H-8), 1.25 (H-9), 1.25 (H-10) and 0.85 ppm (H-11). The last spin system is part of a thiazoline moiety with proton resonances at δ_H_ 5.25 (H-14), δ_H_ 3.69 (H-13a) and δ_H_ 3.63 (H-13b). HMBC correlations from H-13 and H-4 to C-12, in combination with characteristic chemical shift values confirmed the presence of the thiazoline substituent at C-2 of the phenol moiety. The final carbon atom at 171.9 ppm (C-15) could be attributed to a carboxylic acid function with HMBC correlations from H-13 and H-14, thereby completing the determination of the planar structure of **1**. To determine the configuration of **1**, we measured its optical rotation. The obtained value (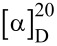 = +37°) was consistent with the value of (*S*)-dihydroaeruginoic acid (**8**, 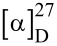 = +47°) which is structurally almost identical to **1** except for the presence of the *n*-pentyl side chain [21]. We thus propose that **1** is also *S*-configured.

Compound **2** (0.4 mg) was obtained as a brown oil. It possesses a molecular ion at *m*/*z* 308.1343 [M + H]^+^, which is consistent with a molecular formula of C_16_H_21_NO_3_S (calcd. for C_16_H_22_NO_3_S, 308.1326) and seven degrees of unsaturation. The NMR data are comparable with compound **1**, except for the presence of an additional carbon atom at δ_C_ 52.3 ppm (C-16) and a proton resonance at δ_H_ 3.73 ppm (H-16). Both H-16 and H-14 show HMBC correlations to the carbonyl C-15. It can therefore be concluded that a methyl ester function replaced the carboxylic acid function of compound **1**. Measurements of the optical rotation of **2** were not possible due to the low quantity of isolated material.

Compound **3** (0.8 mg) was obtained as a brown oil. It possesses a molecular ion at *m*/*z* 292.1017 [M + H]^+^, which suggests a molecular formula of C_15_H_17_NO_3_S (calcd. for C_15_H_18_NO_3_S, 292.1013) and corresponds to eight degrees of unsaturation. A key distinction between compounds **1** and **3** is the lack of two proton signals associated with the thiazoline moiety. Instead, the proton spectrum of **3** features a signal at δ_H_ 8.55 (H-13). The HMBC correlations linking H-13 to 163.3 ppm (C-12) and 146.3 ppm (C-14) indicate the presence of a thiazole rather than a thiazoline moiety. This aligns well with the observed increase in the degree of unsaturation and the reduction in mass by 2 Da.

Compound **4** (0.5 mg) was obtained as a brown oil. It possesses a molecular ion at *m*/*z* 276.0985 [M + H]^+^, which suggests a molecular formula of C_15_H_17_NO_2_S (calcd. for C_15_H_18_NO_3_S, 276.1002) and is in accordance with eight degrees of unsaturation. The NMR data for **4** is closely related with that of compound **3**. The carbon atom at 184.9 ppm (C-15) possesses an HSQC correlation to a proton resonance at δ_H_ 9.98 (H-15), which is characteristic of an aldehyde function. This is in full accordance with the loss of 16 Da compared to compound **3**, confirming that it is the reduced form of the carboxylic acid.

Compound **5** (0.6 mg) was obtained as a brown oil. It possesses a molecular ion at *m*/*z* 278.1231 [M + H]^+^, which suggests a molecular formula of C_15_H_19_NO_2_S (calcd. for C_15_H_20_NO_3_S, 278.1220) and corresponds to seven degrees of unsaturation. The NMR data of compound **5** are very similar to those of compounds **3** and **4**. However, instead of the aldehyde function seen in compound **4**, a proton signal at δ_H_ 4.63 (H-15) can be observed. Together with its distinct carbon signal at 60.3 ppm (C-15), HMBC correlations to the carbon atoms C-13 and C-14 of the thiazole moiety, the loss of one degree of unsaturation and a loss of 2 Da in mass, it can be deduced that compound **5** possesses an alcohol function.

Compound **6** (0.4 mg) was obtained as a brown oil. It possesses a molecular ion at *m*/*z* 291.1165 [M + H]^+^, which suggests a molecular formula of C_15_H_18_N_2_O_2_S (calcd. for C_15_H_19_N_2_O_2_S, 291.1173) and corresponds to eight degrees of unsaturation. In comparison to the other five molecules compound **6** must contain an even number of nitrogen atoms. The NMR data is comparable to compound **3**, but includes a deuterium exchangeable proton signal at δ_H_ 7.62 ppm characteristic for an amide.

In vitro tests were conducted for all compounds to assess their antibacterial activities against three Gram-negative bacteria (*Escherichia coli*, *Agrobacterium tumefaciens* and *Pseudomonas fluorescens*) and one Gram-positive bacterium (*Bacillus subtilis*). The filter paper method was used to determine the inhibition zone diameters, which are reported in Supporting Information File 1, Table S1. It turned out that the compounds possess only modest inhibitory activities against *B. subtilis*. These results are consistent with previous studies [22–23]. Natural products that are structurally related to **1**–**6** were discovered in *Pseudomonas aeruginosa*. It was shown that these compounds function as signaling molecules involved in quorum sensing and stress response [24] which might be an explanation for their low bioactivity against the tested bacteria.

Upon analyzing the structures of the isolated metabolites, it became evident that the predicted lipopeptide siderophore [17] had not been produced by *Massilia* sp. NR 4-1 under the chosen cultivation conditions, as no further extract fraction possessed CAS activity. The reasons for the absence of this compound are not clear. According to the literature, the occurrence of multiple siderophore pathways in a single bacterium is not unusual. Furthermore, it has been repeatedly observed that not all siderophores of such a bacterium must be produced at the same time [25–27]. An illustrative example is the pathogen *P. aeruginosa*, which is capable of adapting its iron acquisition strategy. In general, *P. aeruginosa* relies on a comparatively weak iron chelator named pyochelin. During acute infections, however, when iron availability is severely limited, *P. aeruginosa* switches to the production of a high-affinity siderophore, which further acts as a signal molecule for the production of virulence factors [26]. Similar to *P. aeruginosa*, the causative agent of splenic fever, *Bacillus anthracis*, is also known to synthesize two structurally different siderophores. The corresponding compounds were found to underlie discrete regulation mechanisms, which explains their context-dependent production [27]. The examples of *P. aeruginosa* and *B. anthracis* suggest that siderophore biosynthesis sometimes requires specific triggers beyond iron deficiency, which may not be met under laboratory conditions.

**Figure 4:**
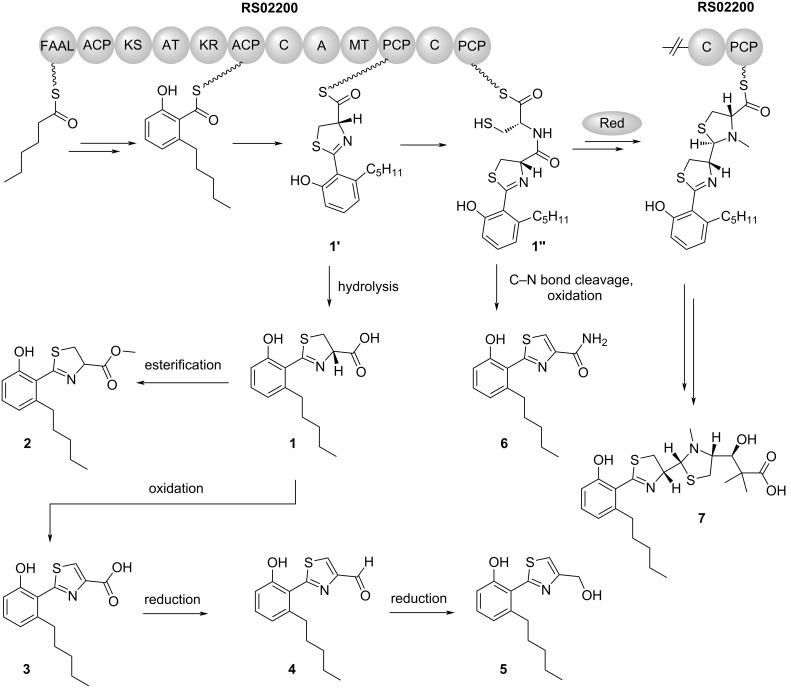
Proposed origin of the isolated compounds **1–6** as well as massiliachelin (**7**). Domain notation of the protein RS02200: FAAL: fatty acyl-AMP ligase; ACP: acyl carrier protein; KS: β-ketoacyl synthase; AT: acyltransferase; KR: ketoreductase; C: condensation; A: adenylation; MT: methyltransferase; PCP: peptidyl carrier protein. A discrete enzyme, the thiazolinyl imide reductase RS02195 (Red), catalyzes the conversion of a thiazoline into a thiazolidine ring.

The six metabolites that were recovered in this study share a phenolic moiety with a thiazole or thiazoline substituent. This motif is present in many siderophores, e.g., in pyochelin [26], yersiniabactin [28], agrochelin [29], micacocidin [30], the *Massilia*-derived massiliachelin [18], as well as in piscibactin [31] and the photoxenobactins [32]. A unifying theme in the biosynthesis of these natural products is the use of a thiotemplate-based assembly strategy [33]. The molecular building blocks that are needed for the biosynthesis are covalently bound via thioester bonds to multi-domain enzymes. The domains fulfill specific functions, ranging from the selection and linkage of building blocks to their chemical modification. A plausible scenario for the formation of compounds **1**–**6** involves the enzymatic machinery for massiliachelin biosynthesis, namely the protein RS02200 [18]. According to our proposal (Figure 4), the biosynthesis starts from hexanoic acid which, upon its thioesterification, is elongated by three decarboxylative Claisen condensations with malonyl-CoA to a 6-pentylsalicyl thioester. A condensation with cysteine and a subsequent cyclization generate a 6-pentylsalicyl-thiazolinyl thioester intermediate (**1’**). In massiliachelin biosynthesis this intermediate is further processed and elongated with another cysteine-derived thiazoline, which is eventually reduced [18]. A premature hydrolytic release of **1’** from the assembly line would give **1**, which could be further modified to **2**–**5**. Some corresponding reactions (e.g., hydrolysis, esterification, oxidation) might be due to the isolation conditions or they could be attributed to unspecific enzymatic biotransformations. For compound **1**, no spontaneous conversion to the ester **2** was observed, even after storage in methanol for two months. In contrast, the formation of the terminal carboxamide in **6** might be due to a spontaneous C–N bond cleavage, which occurs in **1’’** prior to the cyclization, consistent with a mechanism recently proposed in photoxenobactin biosynthesis [34].

Despite the widespread occurrence of siderophores featuring a phenolic moiety with a thiazole or thiazoline substituent, compounds **2**–**6** have not been reported before according to literature searches. Compound **1** was already described as an intermediate in the chemical total synthesis of micacocidin [35], but has not been isolated from a natural source before.

## Conclusion

In summary, six metabolites that are structurally related to the siderophore massiliachelin were recovered from a culture extract of *Massilia* sp. NR 4-1. The structures of the natural products were verified by high-resolution mass spectrometry as well as 1D and 2D NMR analyses. The newly found compounds are assumed to represent intermediates or shunt products in massiliachelin biosynthesis. A model for their formation is presented.

## Experimental

### Analytical methods

Preparative HPLC was conducted on a Shimadzu LC-20A system equipped with two pumps (LC-20AD), a photo-diode array detector (SPD-M20A), a degasser unit (DGU-20A), an automatic sampler (SIL-20A), and a column oven (CTO-20AC). LC–MS analysis was performed on a compact quadrupole-time of flight (Q-TOF) mass spectrometer from Bruker Daltonics with an Agilent 1260 Infinity LC system equipped with a Nucleoshell RP18 column (150 × 2.0 mm, 5 µm, Macherey-Nagel). NMR spectra were recorded on a Bruker 600 MHz Avance III HD system with DMSO-*d*_6_ as solvent and internal standard. The solvent signal was referenced to δ_H_ 2.50 ppm and δ_C_ 39.52 ppm, respectively. Optical rotation was measured at 20 °C on a PerkinElmer polarimeter 341 with a sodium lamp (wavelength = 589 nm) using a 1 dm cuvette. For this, samples were dissolved in 1 mL methanol.

### Cultivation and extraction of *Massilia* sp. NR 4-1

For metabolite production, the strain was grown in 5 L Erlenmeyer flasks containing 1.5 L of modified R2A medium: 0.5 g/L yeast extract, 0.5 g/L proteose peptone, 0.5 g/L casamino acids, 0.5 g/L glucose, 0.5 g/L soluble starch, 0.3 g/L KH_2_PO_4_, 0.05 g/L MgSO_4_ × 7 H_2_O. The pH of the medium was adjusted to pH 7.2. The cultivation was conducted on a rotary shaker at 130 rpm and 30 °C for one week. Afterwards adsorber resin (XAD-7, 20 g/L) was added to the culture broth to bind the secreted metabolites. The resin was separated from the culture broth by filtration, washed with distilled water and exhaustively extracted with methanol.

### Isolation of derivatives

The concentrated extract was first fractionated by reversed-phase HPLC using a Nucleodur C18 Isis column (250 × 10 mm, 5 μm, Macherey-Nagel) and a gradient of acetonitrile in water supplemented with 0.1% (v/v) trifluoroacetic acid. The gradient conditions were as follows: from 20% acetonitrile to 90% in 30 minutes and kept at 90% for 10 minutes. The flow rate was set to 5 mL/min. The elution of compounds was monitored with a diode array detector over the range from 190 to 650 nm. Subsequently, the relevant metabolites were further purified by isocratic fractionation, by lowering the concentration of acetonitrile by 10% compared to the elution concentration that was achieved with the gradient method.

### Chrome azurol S assay

The CAS assay solution was prepared according to a previously reported protocol [20]. Briefly, chrome azurol S (CAS) and hexadecyltrimethylammonium bromide (HDTMA) were independently dissolved in water to prepare 2 mM and 6.585 M solutions, respectively. In a beaker, 7.5 mL of the CAS solution were mixed with 15 mL of the HDTMA solution under stirring. To this mixture, 50 mL of water, 1.5 mL of an iron chloride solution (1 mM FeCl × 6 H_2_O in 10 mM HCl), 4.3 g of anhydrous piperazine and 6.25 mL of 12 M hydrochloric acid were added. The resulting mixture was then diluted with water to a final volume of 100 mL, hence yielding the blue CAS assay solution. To perform the assay, 1 mL of the CAS assay solution was pipetted into a cuvette followed by the addition of 0.2 mL of the substance to be tested. A color change from blue to yellow indicated the presence of metal-chelating molecules.

### Filter paper assay

The bacterial strains were acquired from the Leibniz Institute DSMZ (German Collection of Microorganisms and Cell Cultures GmbH). To test antibacterial activity, 0.1 mL of an overnight culture of each bacterial strain was plated on agar plates containing their preferred medium. The samples being tested were dissolved in methanol at a concentration of 50 µg/10 µL. Each compound was impregnated onto filter papers at a volume of 10 µL per disc. The inoculated plates were then incubated at 30 °C for 48 hours. The antibacterial activity was evaluated by measuring the zone of inhibition against the test organism. Ampicillin (Roth, Carl Roth GmbH + Co. KG, Germany), tetracycline (Fluka Honeywell International Inc., United States of America) and ciprofloxacin (Sigma, Sigma-Aldrich Chemie GmbH, Germany) were used as positive controls.

## Supporting Information

File 1 UV and total ion chromatograms of culture extracts from *Massilia* sp. NR 4-1. Copies of MS/MS and NMR spectra for new compounds.
