# Supplementary material for: Intermediates and shunt products of massiliachelin biosynthesis in Massilia sp. NR 4-1
Source: Beilstein J Org Chem. 2023 Jun 23;19:909–17. doi: 10.3762/bjoc.19.69 (PMC10291242; doi:10.3762/bjoc.19.69)
Supplement: File 1 — UV and total ion chromatograms of culture extracts from Massilia sp. NR 4-1. Copies of MS/MS and NMR spectra for new compounds. [file Beilstein_J_Org_Chem-19-909-s001.pdf]

BEILSTEIN JOURNAL OF ORGANIC CHEMISTRY

Supporting Information

for

Intermediates and shunt products of massiliachelin biosynthesis in *Massilia* sp. NR 4-1

Till Steinmetz, Blaise Kimbadi Lombe and Markus Nett

Beilstein J. Org. Chem. **2023,** *19,* 909–917. doi:10.3762/bjoc.19.69

UV and total ion chromatograms of culture extracts from *Massilia* sp. NR 4-1. Copies of MS/MS and NMR spectra for new compounds

License and Terms: This is a supporting information file under the terms of the Creative Commons Attribution License (https://creativecommons.org/licenses/by/4.0). Please note that the reuse, redistribution and reproduction in particular requires that the author(s) and source are credited and that individual graphics may be subject to special legal provisions.
The license is subject to the *Beilstein Journal of Organic Chemistry* terms and conditions: (https://www.beilstein-journals.org/bjoc/terms)

Table S1: Inhibition zone diameters of **1**-**6** against test bacteria.. S1
Figure S1: UV chromatogram (585 nm) of crude culture extract from *Massilia* sp. NR 4-1 grown with sodium pyruvate (red) and without sodium pyruvate (black). S2
Figure S2: UV chromatogram (280 nm) of the crude culture extract from *Massilia* sp. NR 4-1. ....S3
Figure S3: Total ion chromatogram of the crude culture extract from *Massilia sp*. NR 4-1 ....S4
Figure S4: MS/MS spectrum of **1**...S5
Figure S5: ^{1}H NMR spectrum of **1** (600 MHz, DMSO-d_6, 25 °C) S6
Figure S6: ^{13}C NMR spectrum of **1** (150 MHz, DMSO-d_6, 25 °C) S7
Figure S7: COSY spectrum of **1** (600 MHz, DMSO-d_6, 25 °C) S8
Figure S8: HSQC spectrum of **1** (600 MHz, DMSO-d_6, 25 °C)..S9
Figure S9: HMBC spectrum of **1** (600 MHz, DMSO-d_6, 25 °C)...S10
Figure S10: MS/MS spectrum of **2**...S11
Figure S11: ^{1}H NMR spectrum of **2** (600 MHz, DMSO-d_6, 25 °C) S12
Figure S12: ^{13}C NMR spectrum of **2** (150 MHz, DMSO-d_6, 25 °C) S13
Figure S13: COSY spectrum of **2** (600 MHz, DMSO-d_6, 25 °C) S14
Figure S14: HSQC spectrum of **2** (600 MHz, DMSO-d_6, 25 °C)...S15
Figure S15: HMBC spectrum of **2** (600 MHz, DMSO-d_6, 25 °C)..S16
Figure S16: MS/MS spectrum of **3**...S17
Figure S17: ^{1}H NMR spectrum of **3** (600 MHz, DMSO-d_6, 25 °C) S18
Figure S18: ^{13}C NMR spectrum of **3** (150 MHz, DMSO-d_6, 25 °C) S19
Figure S19: COSY spectrum of **3** (600 MHz, DMSO-d_6, 25 °C) S20
Figure S20: HSQC spectrum of **3** (600 MHz, DMSO-d_6, 25 °C)...S21
Figure S21: HMBC spectrum of **3** (600 MHz, DMSO-d_6, 25 °C)..S22
Figure S22: MS/MS spectrum of **4**...S23
Figure S23: ^{1}H NMR spectrum of **4** (600 MHz, DMSO-d_6, 25 °C) S24
Figure S24: ^{13}C NMR spectrum of **4** (150 MHz, DMSO-d_6, 25 °C) S25
Figure S25: COSY spectrum of **4** (600 MHz, DMSO-d_6, 25 °C) S26
Figure S26: HSQC spectrum of **4** (600 MHz, DMSO-d_6, 25 °C)...S27
Figure S27: HMBC spectrum of **4** (600 MHz, DMSO-d_6, 25 °C)..S28
Figure S28: MS/MS spectrum of **5**...S29
Figure S29: ^{1}H NMR spectrum of **5** (600 MHz, DMSO-d_6, 25 °C) S30
Figure S30: ^{13}C NMR spectrum of **5** (150 MHz, DMSO-d_6, 25 °C) S31
Figure S31: COSY spectrum of **5** (600 MHz, DMSO-d_6, 25 °C) S32
Figure S32: HSQC spectrum of **5** (600 MHz, DMSO-d_6, 25 °C)...S33
Figure S33: HMBC spectrum of **5** (600 MHz, DMSO-d_6, 25 °C)..S34
Figure S34: MS/MS spectrum of **6**...S35
Figure S35: ^{1}H NMR spectrum of **6** (600 MHz, DMSO-d_6, 25 °C) S36
Figure S36: ^{13}C NMR spectrum of **6** (150 MHz, DMSO-d_6, 25 °C) S37
Figure S37: COSY spectrum of **6** (600 MHz, DMSO-d_6, 25 °C) S38
Figure S38: HSQC spectrum of **6** (600 MHz, DMSO-d_6, 25 °C)...S39
Figure S39: HMBC spectrum of **6** (600 MHz, DMSO-d_6, 25 °C)..S40

Table S1**:** Inhibition zone diameters of **1**-**6** as well as the reference antibiotics ampicillin (amp), tetracycline (tet) and ciprofloxacin (cip) against the tested bacteria.

	**Diameter of Inhibition Zone (mm) in 10 yL/disk**			
	B. subtilis (DSM 168)	*E. coli* (DSM 18039)	*P. fluorescens* (DSM 11532)	*A. tumefaciens* (C58)
**1**	2	-	-	1
**2**	-	-	-	-
**3**	4	-	-	2
**4**	3	-	-	-
**5**	3	-	-	-
**6**	0.5	-	-	0.5
**ampicillin**	14	22	-	22
**tetracycline**	22	20	16	11
**ciprofloxacin**	37	38	24	31

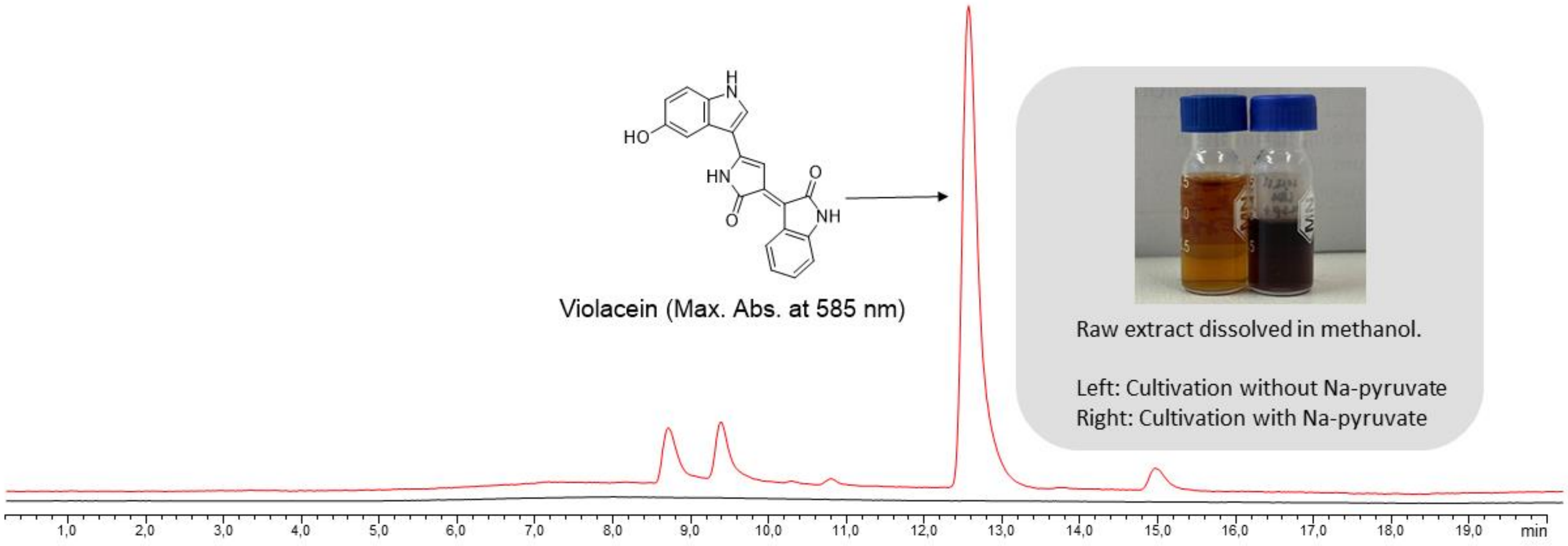

Figure S1: UV chromatogram (585 nm) of crude culture extract from *Massilia* sp. NR 4-1 grown with sodium pyruvate (red) and without sodium pyruvate (black). The darker coloring of the raw extract is due to a higher concentration of violacein.

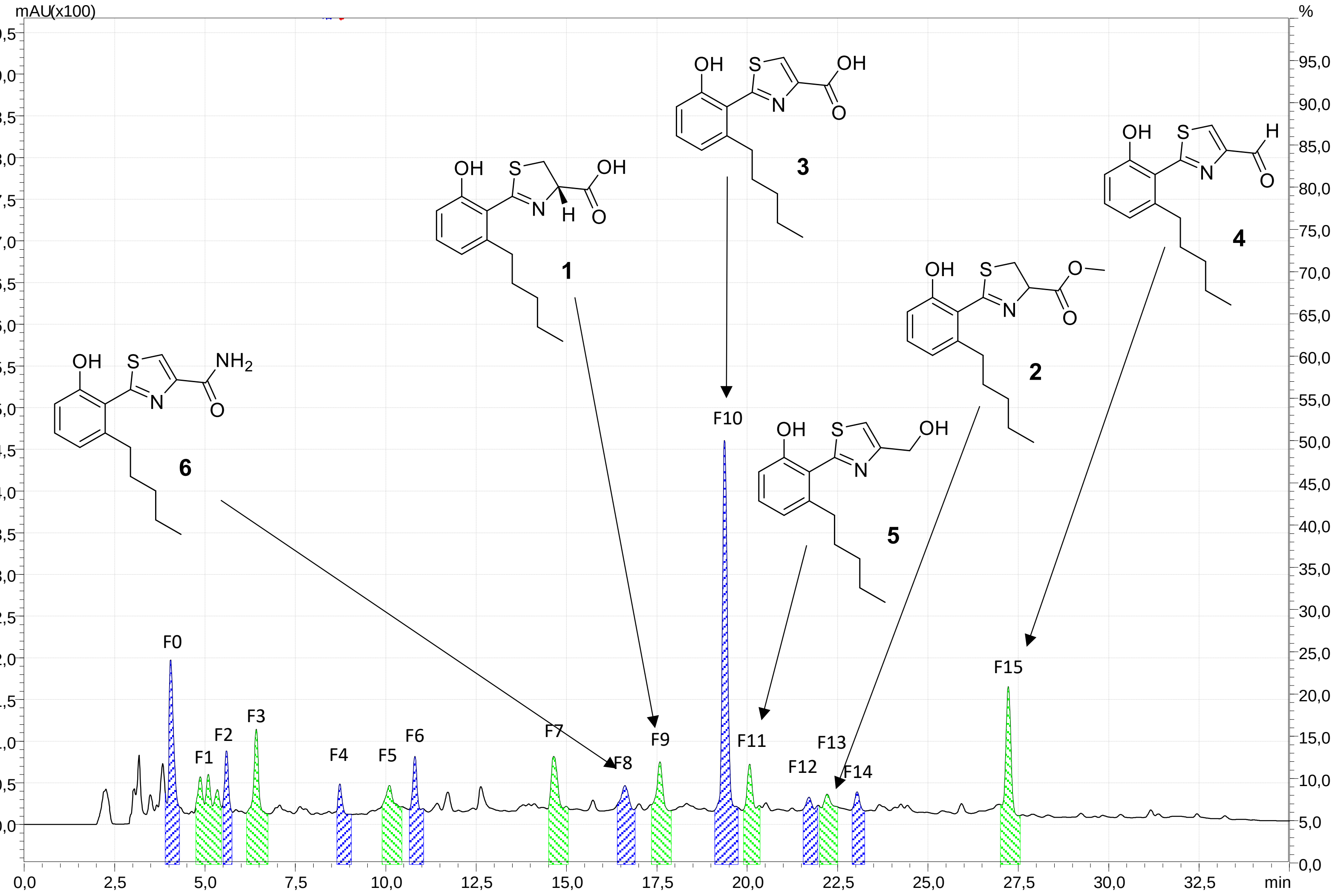

Figure S2: UV chromatogram (280 nm) of the crude culture extract from *Massilia* sp. NR 4-1. Fractions 8, 9, 10, 11, 13, and 15 showed a visible color change with the CAS assay from blue to yellow.

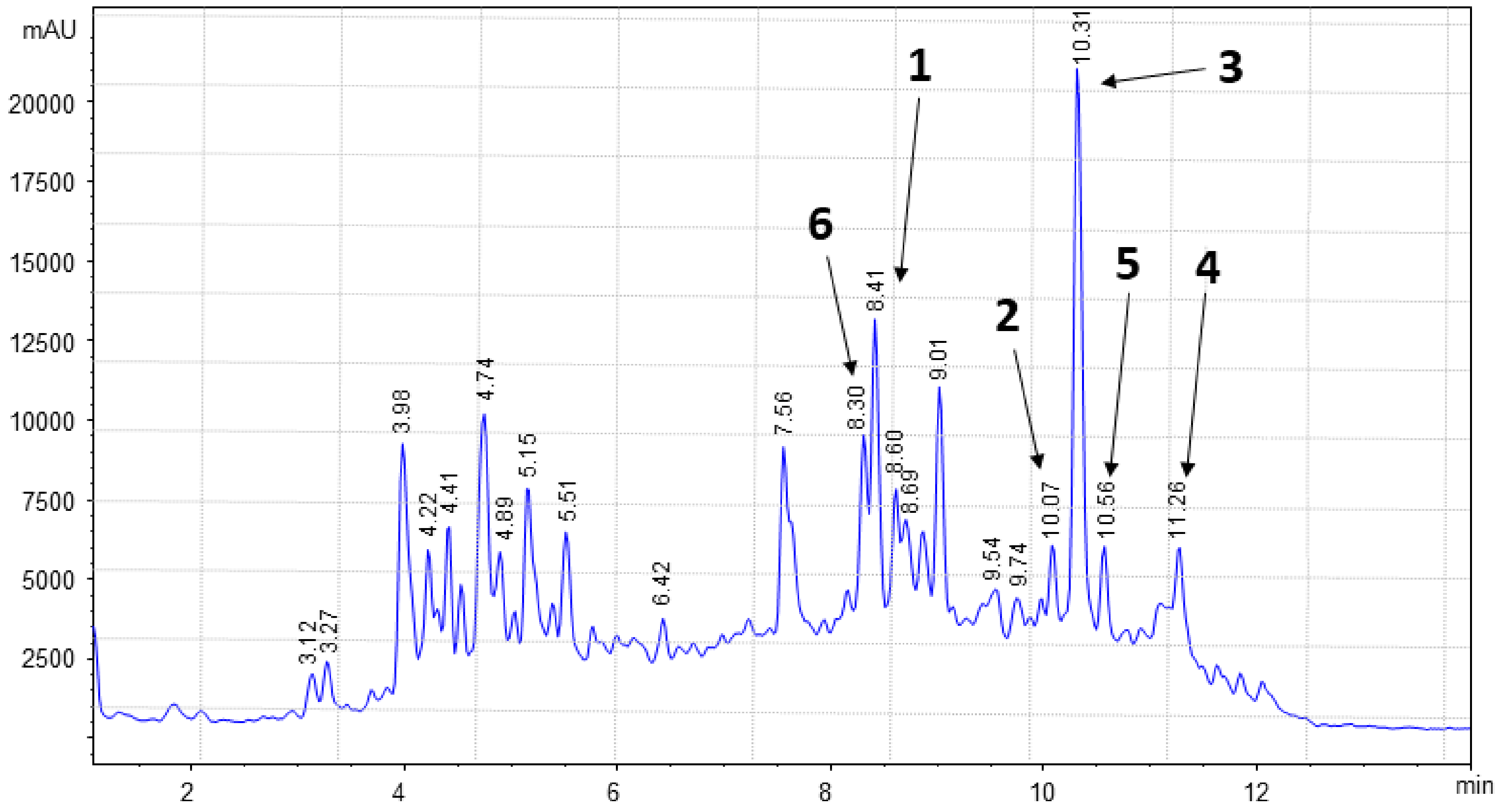

Figure S3: Total ion chromatogram of the crude culture extract from *Massilia sp.* NR 4-1.

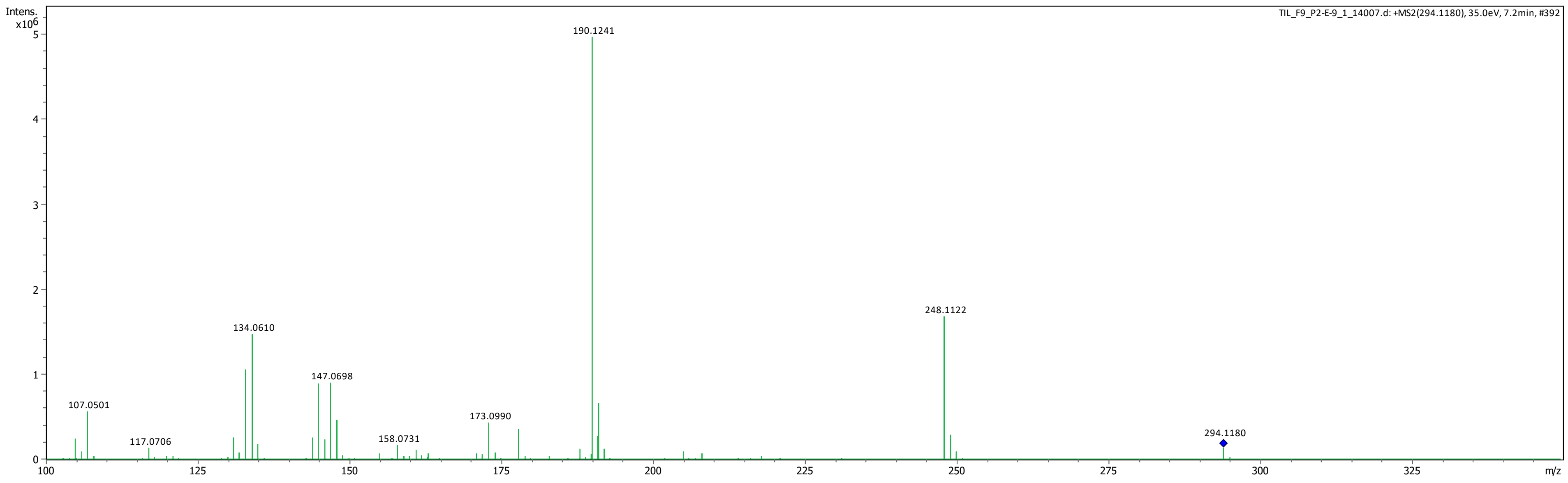

Figure S1: MS/MS spectrum of **1**

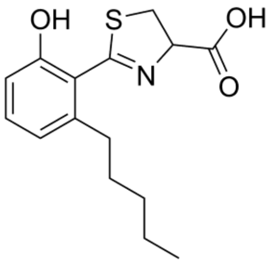

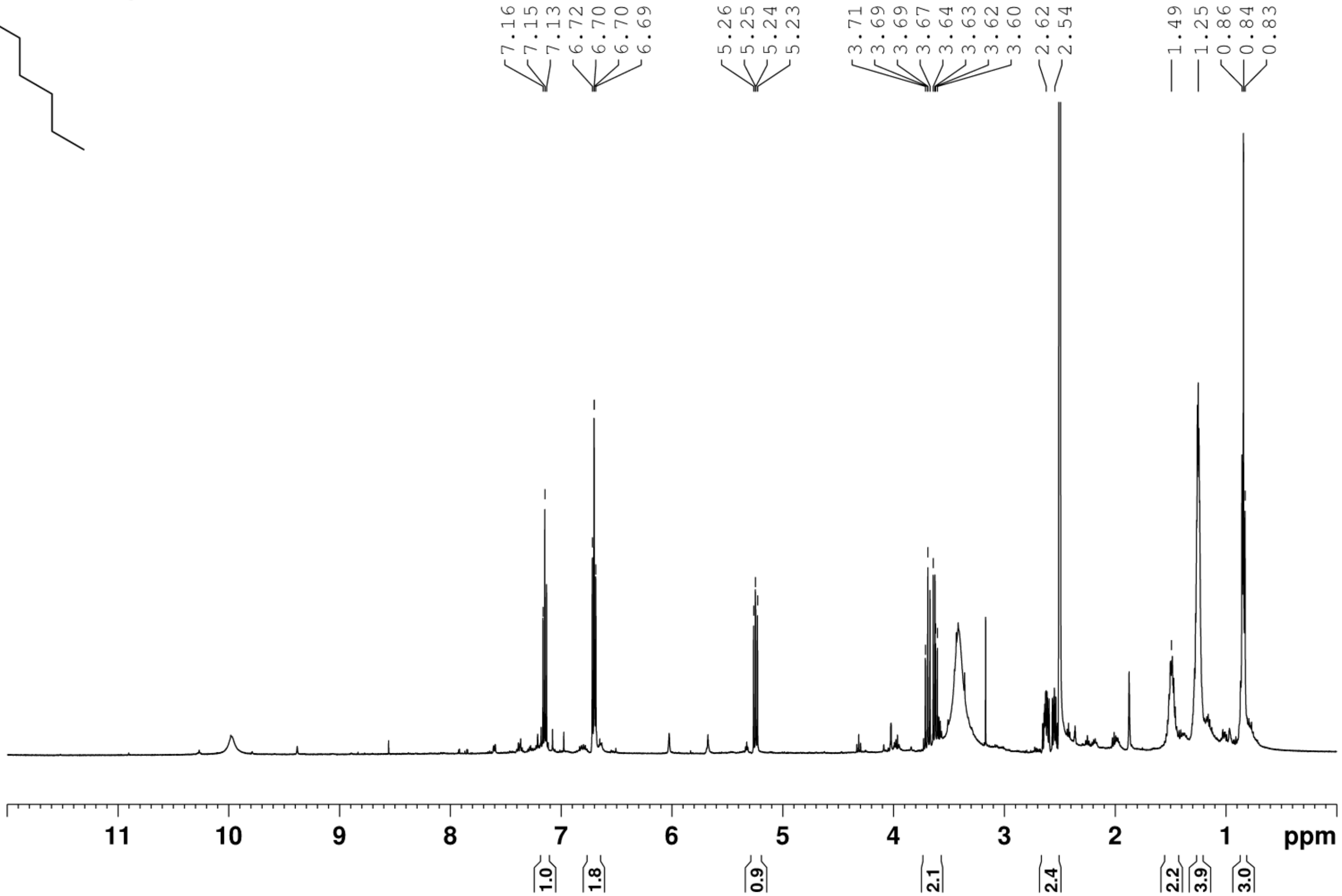

Figure S2: ^{1}H NMR spectrum of **1** (600 MHz, DMSO-d_6, 25 °C)

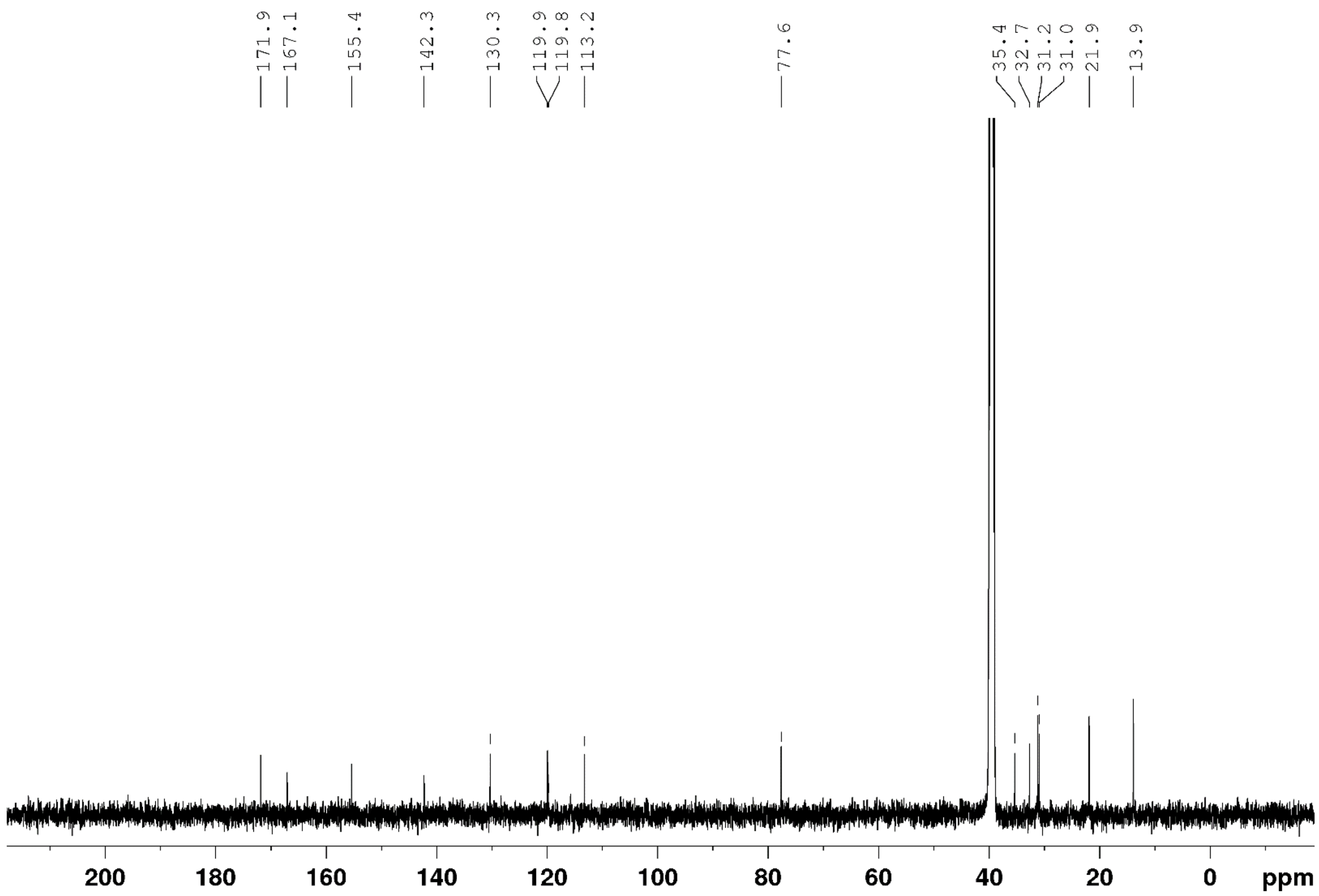

Figure S3: ^{13}C NMR spectrum of **1** (150 MHz, DMSO-d_6, 25 °C)

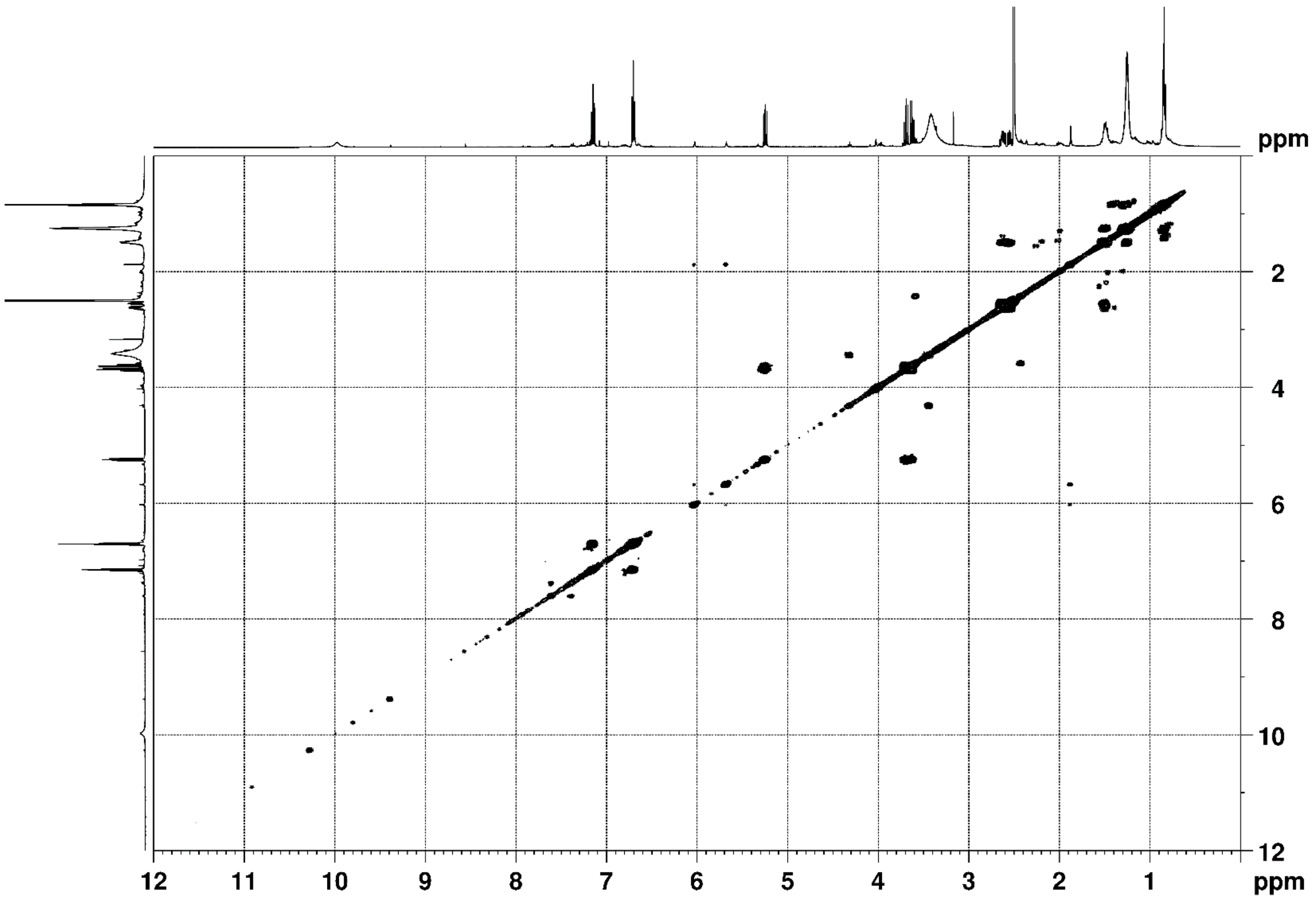

Figure S4: COSY spectrum of **1** (600 MHz, DMSO-d_6, 25 °C)

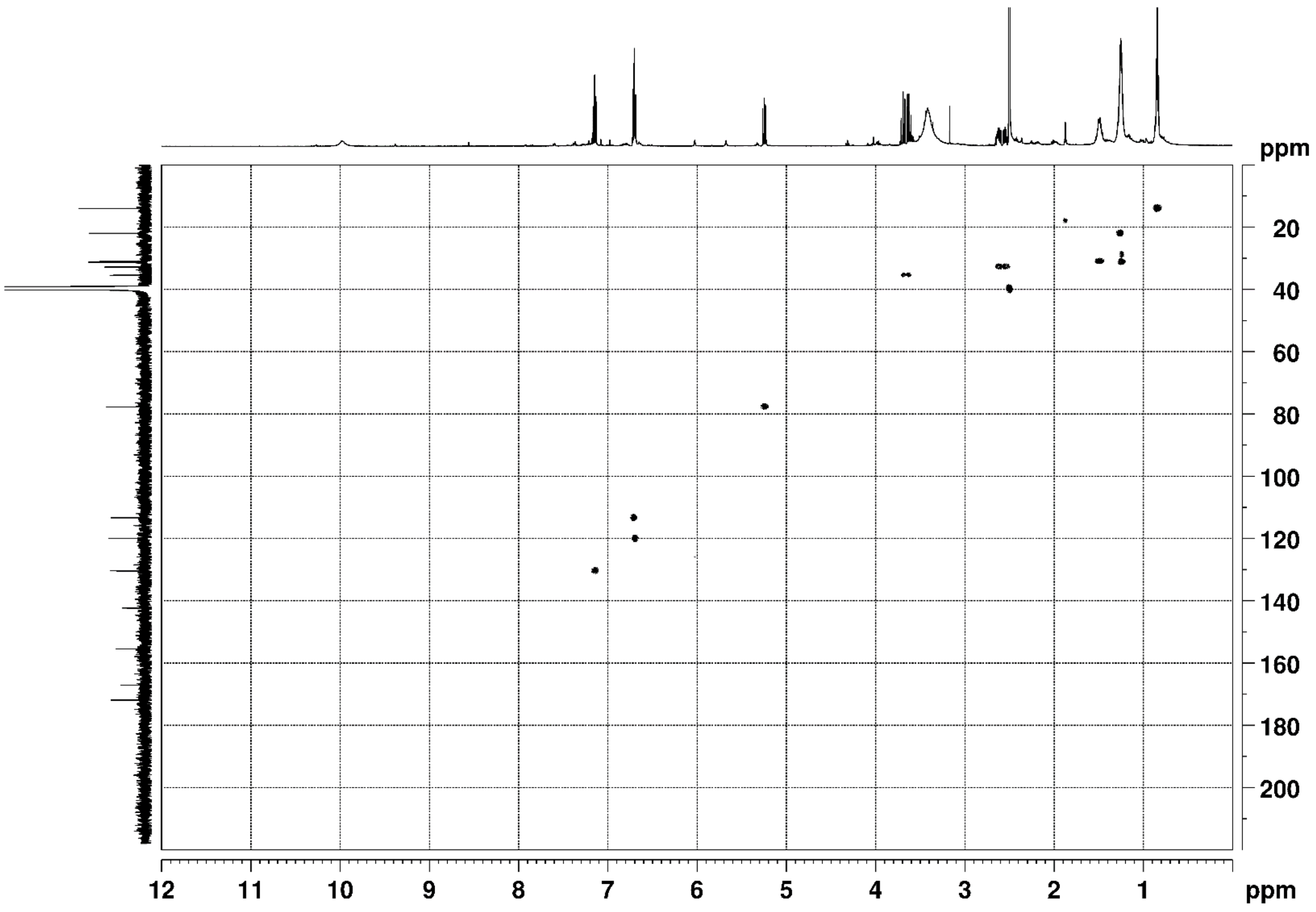

Figure S5: HSQC spectrum of **1** (600 MHz, DMSO-d_6, 25 °C)

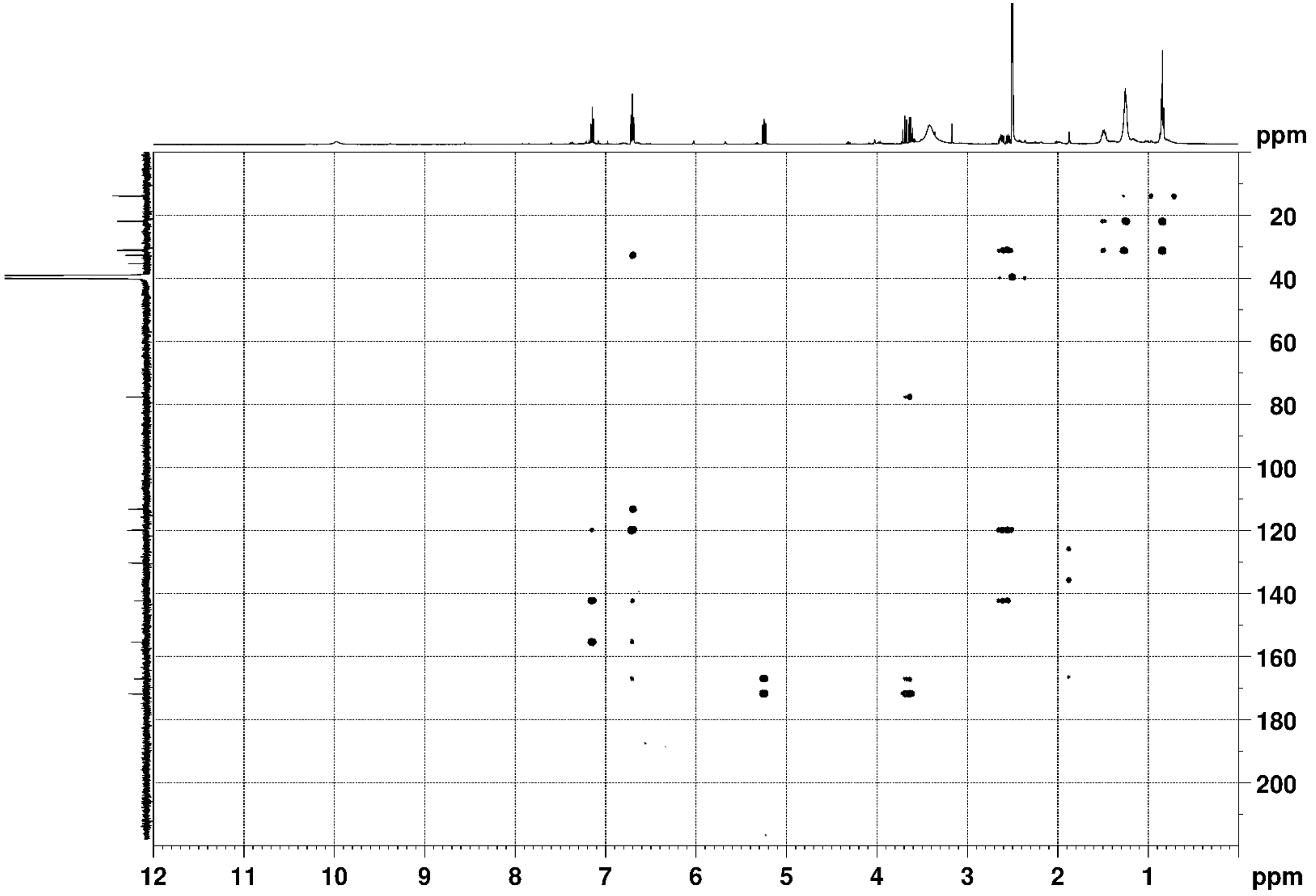

Figure S6: HMBC spectrum of **1** (600 MHz, DMSO-d_6, 25 °C)

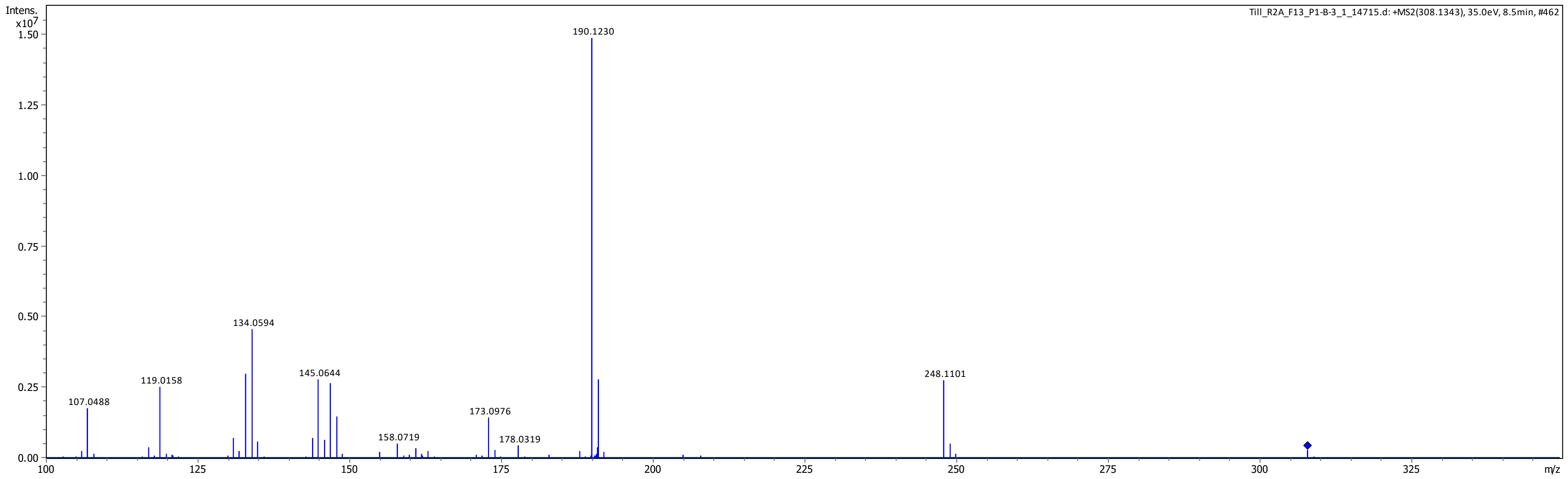

Figure S7: MS/MS spectrum of **2**

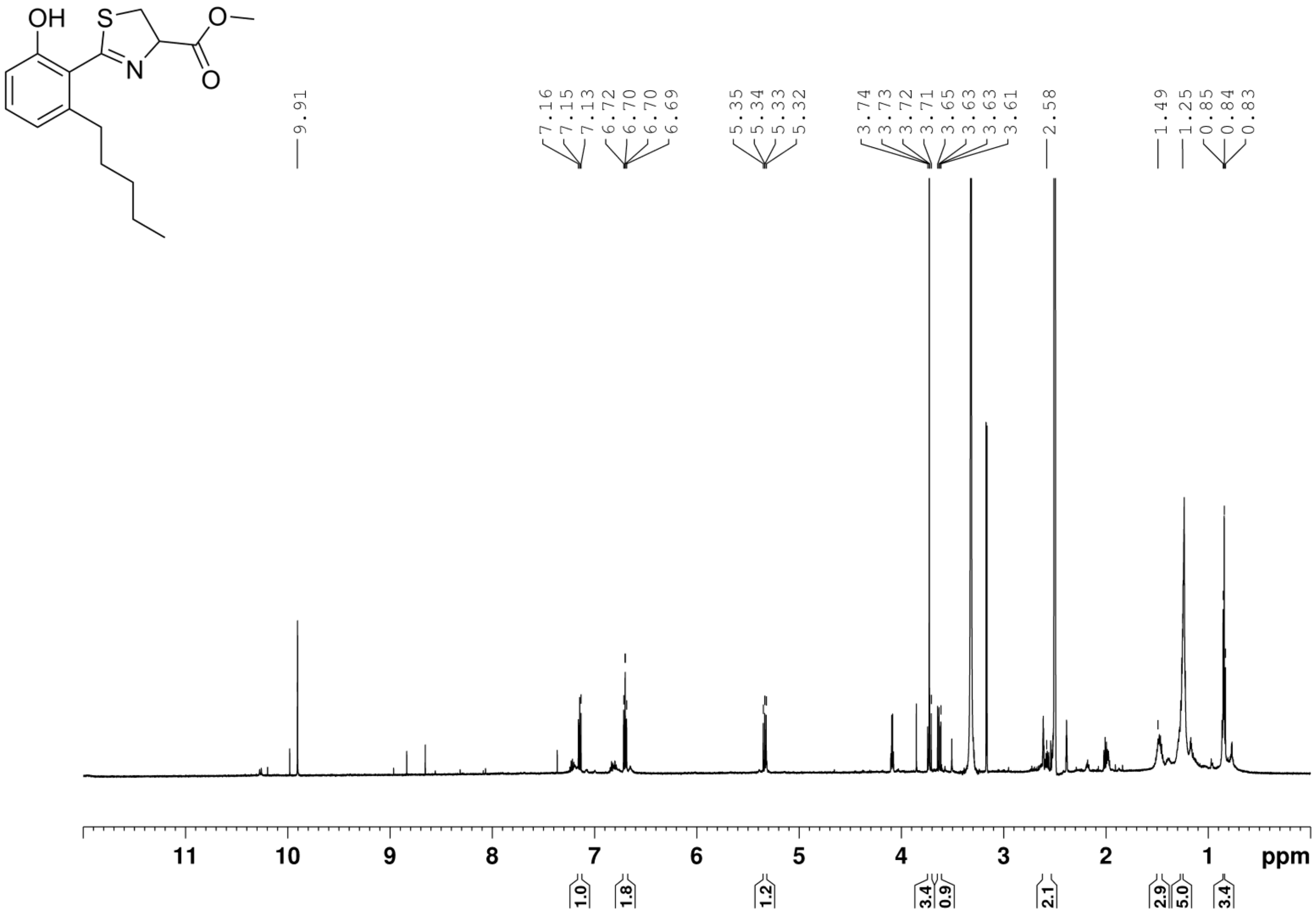

Figure S8: ^{1}H NMR spectrum of **2** (600 MHz, DMSO-d_6, 25 °C)

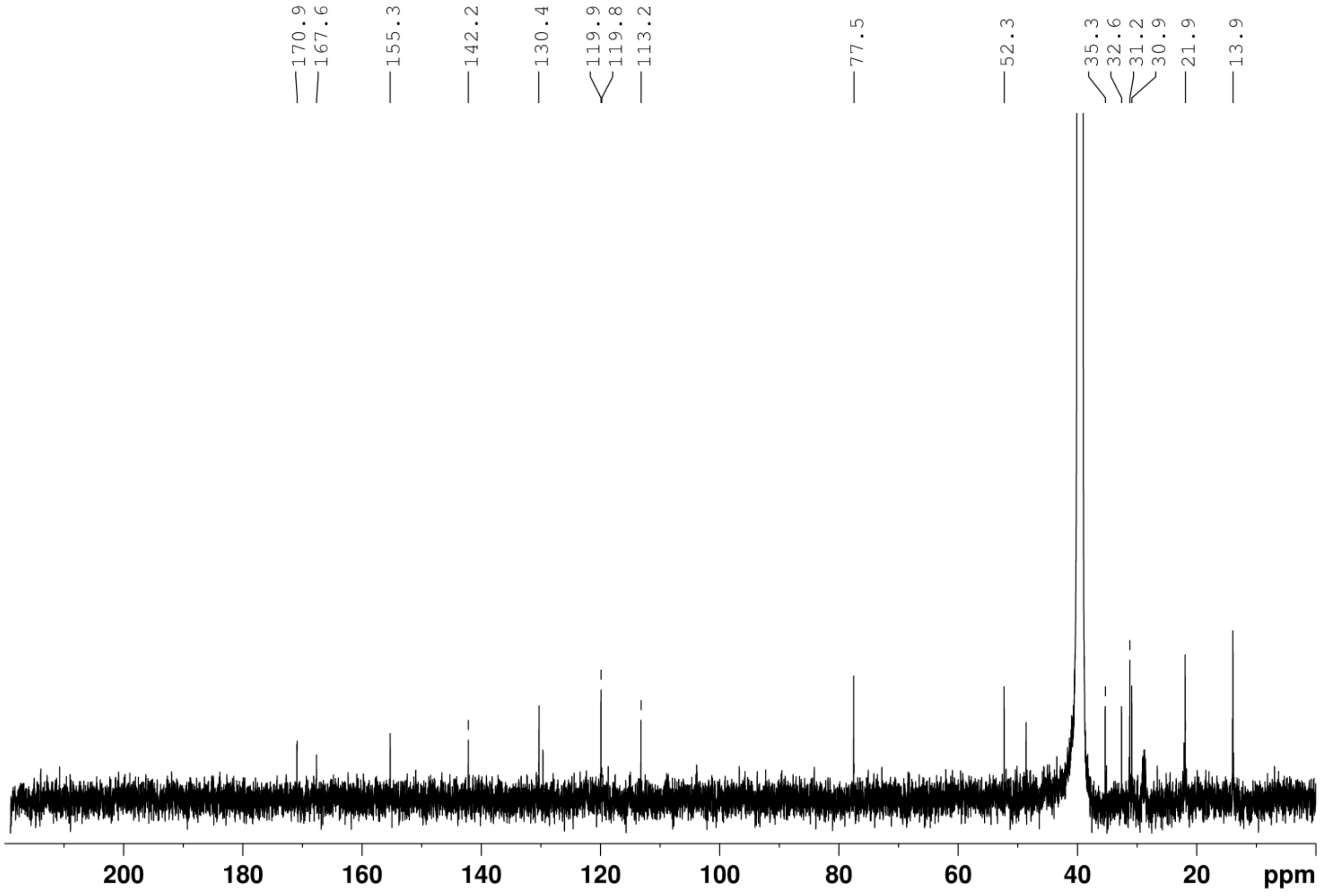

Figure S9: ^{13}C NMR spectrum of **2** (150 MHz, DMSO-d_6, 25 °C)

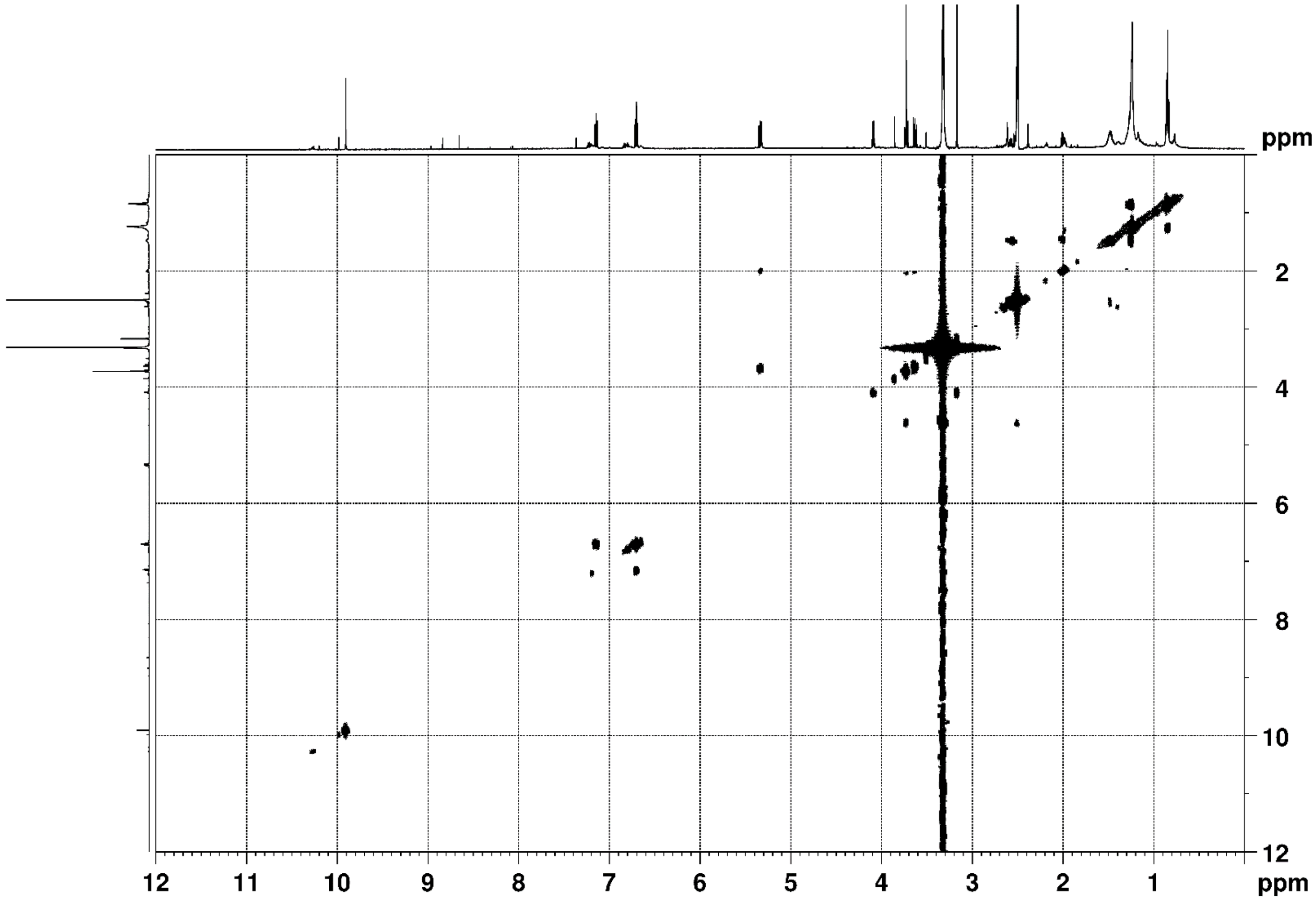

Figure S10: COSY spectrum of **2** (600 MHz, DMSO-d_6, 25 °C)

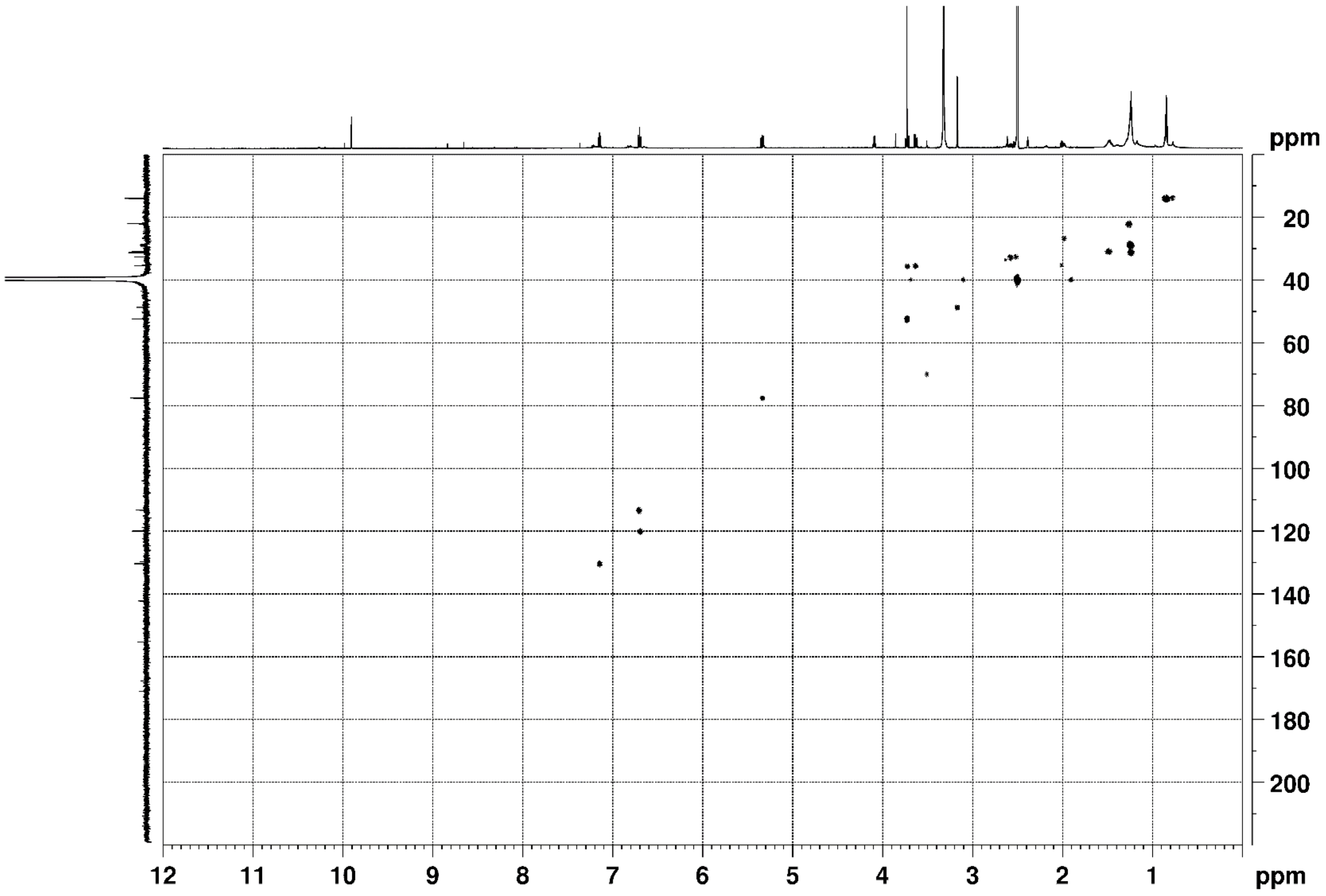

Figure S11: HSQC spectrum of **2** (600 MHz, DMSO-d_6, 25 °C)

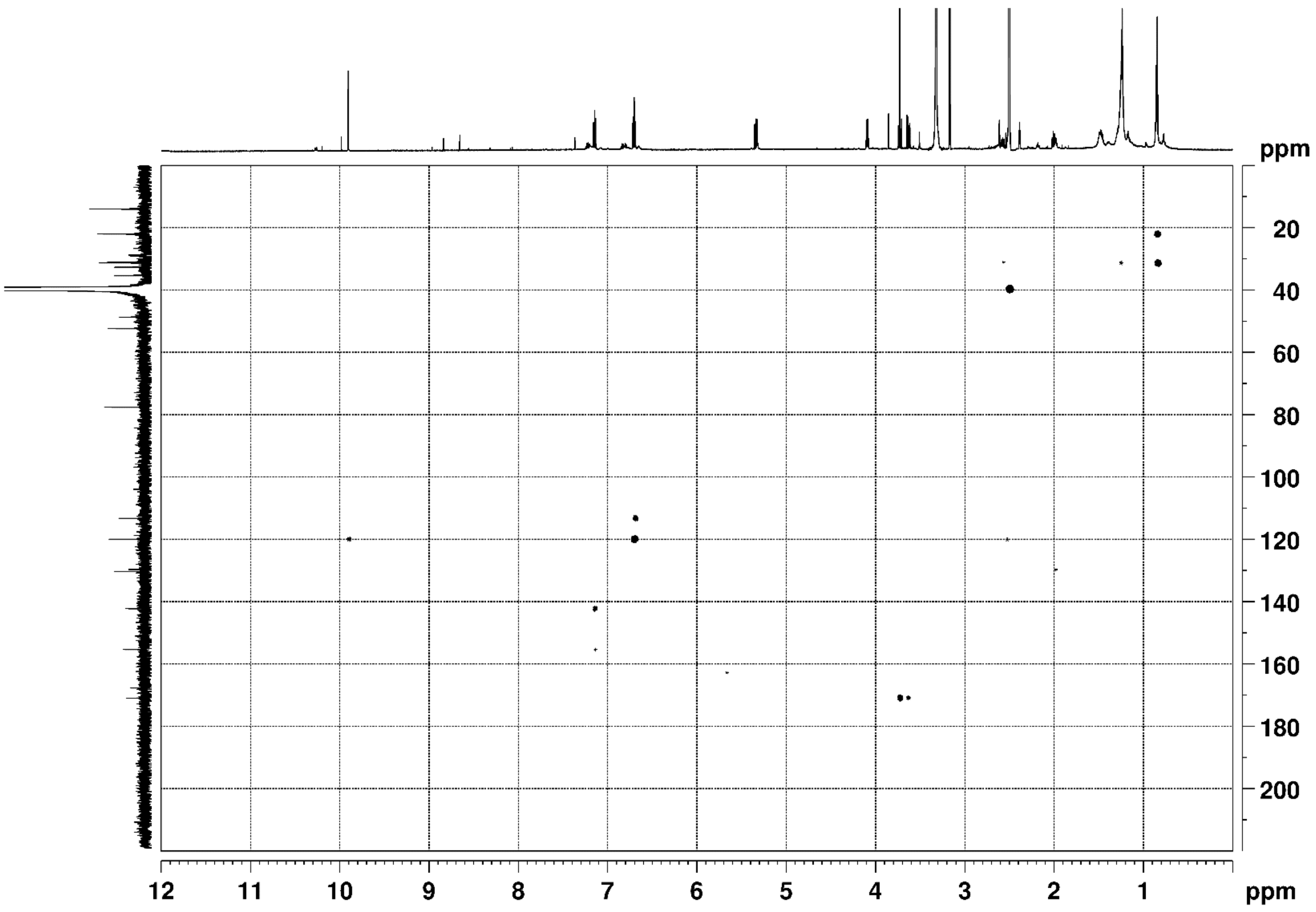

Figure S12: HMBC spectrum of **2** (600 MHz, DMSO-d_6, 25 °C)

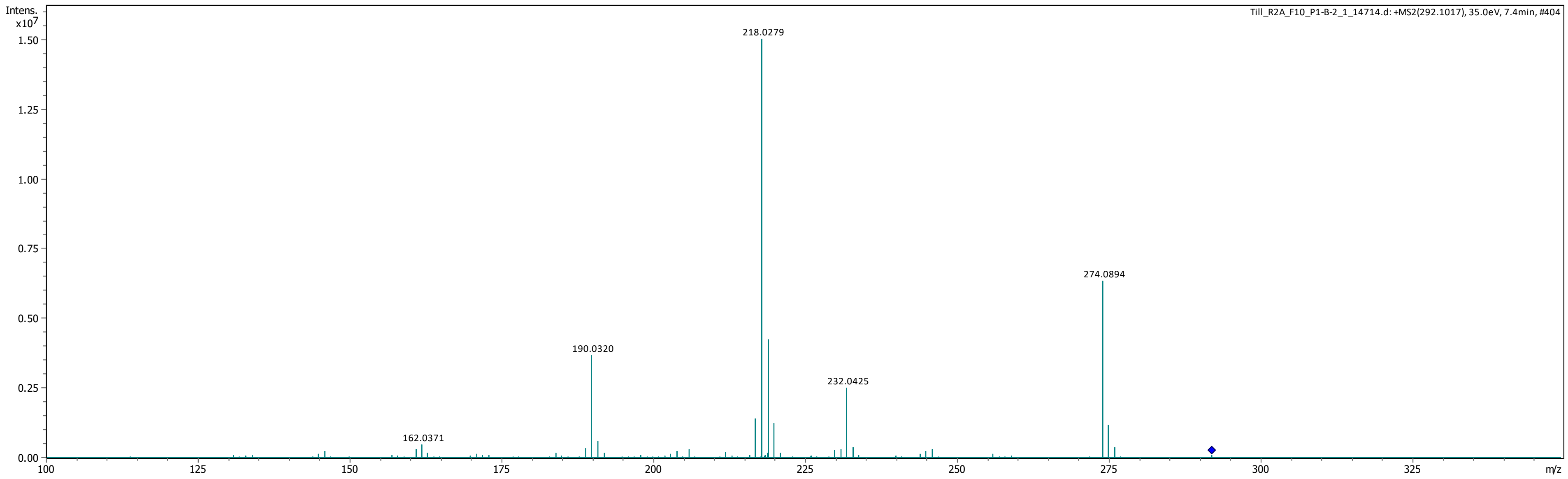

Figure S13: MS/MS spectrum of **3**

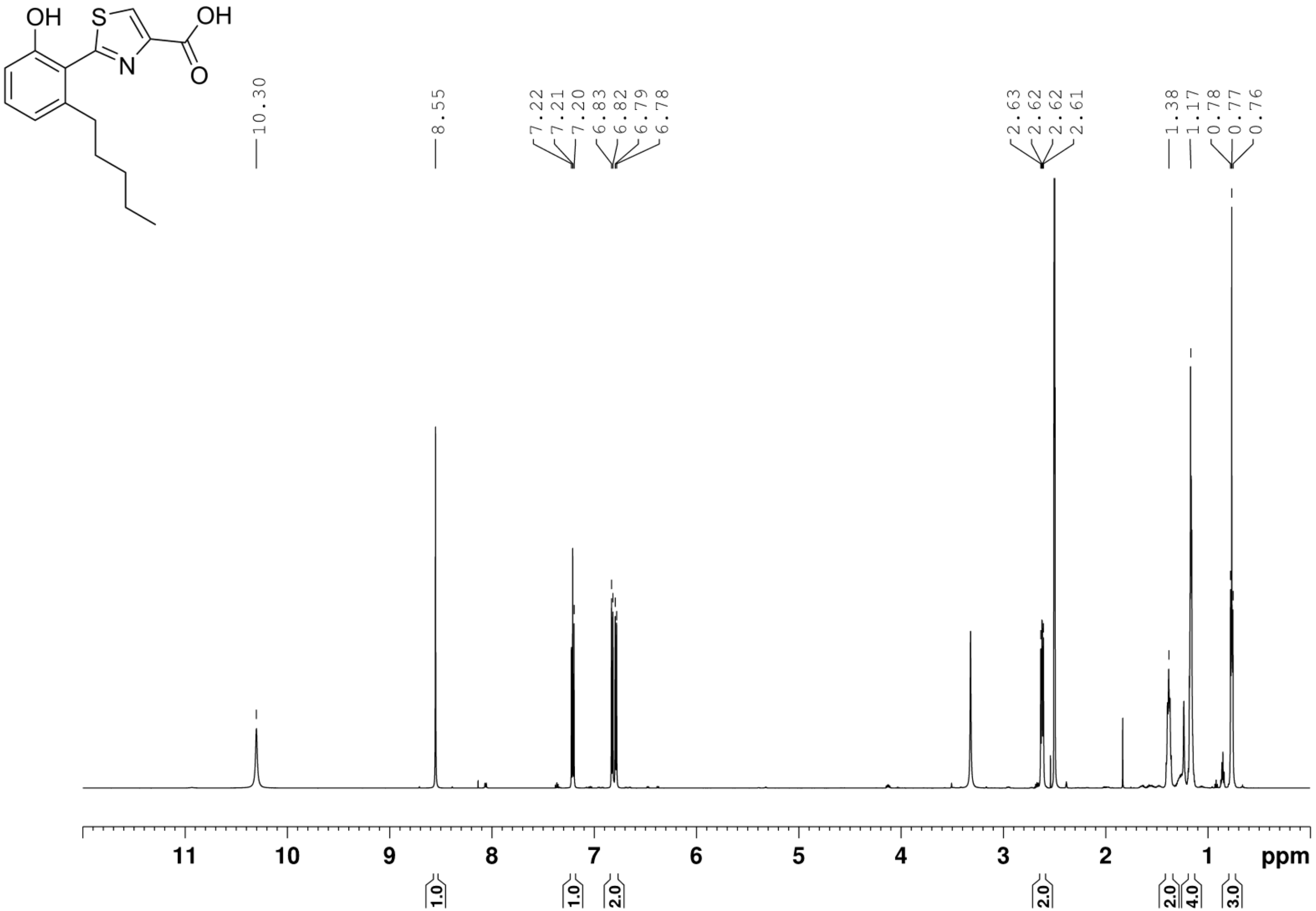

Figure S14: ^{1}H NMR spectrum of **3** (600 MHz, DMSO-d_6, 25 °C)

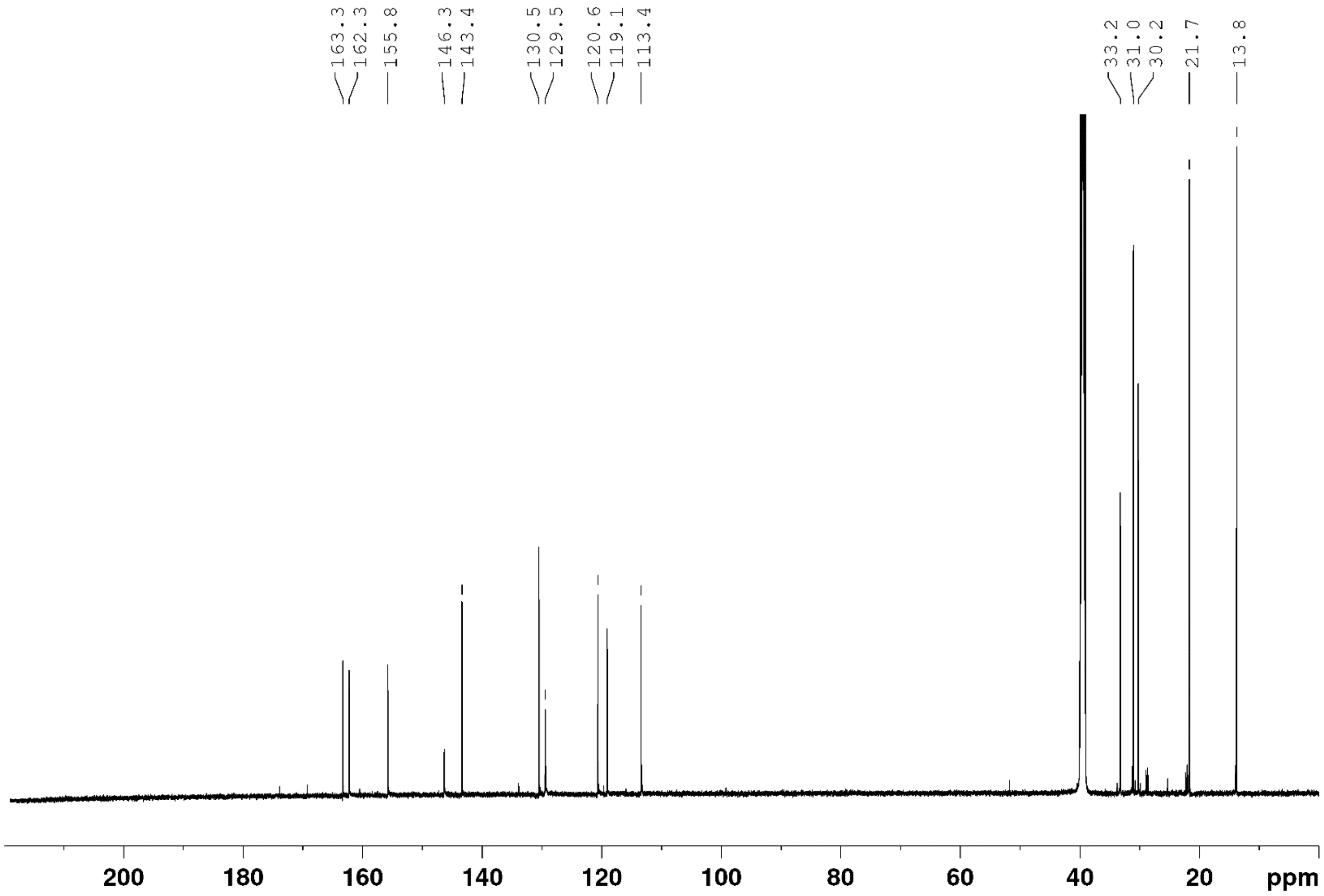

Figure S15: ^{13}C NMR spectrum of **3** (150 MHz, DMSO-d_6, 25 °C)

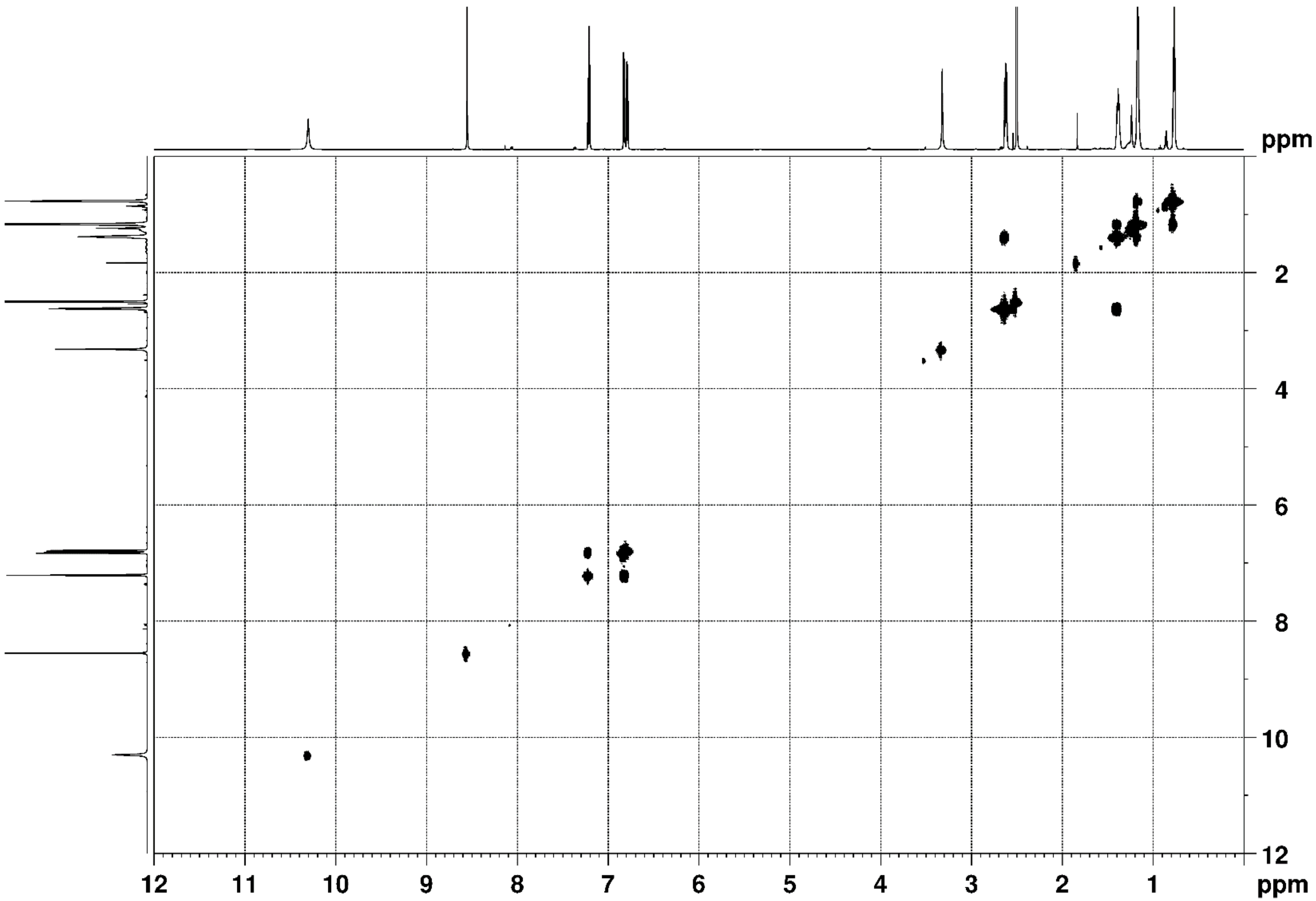

Figure S16: COSY spectrum of **3** (600 MHz, DMSO-d_6, 25 °C)

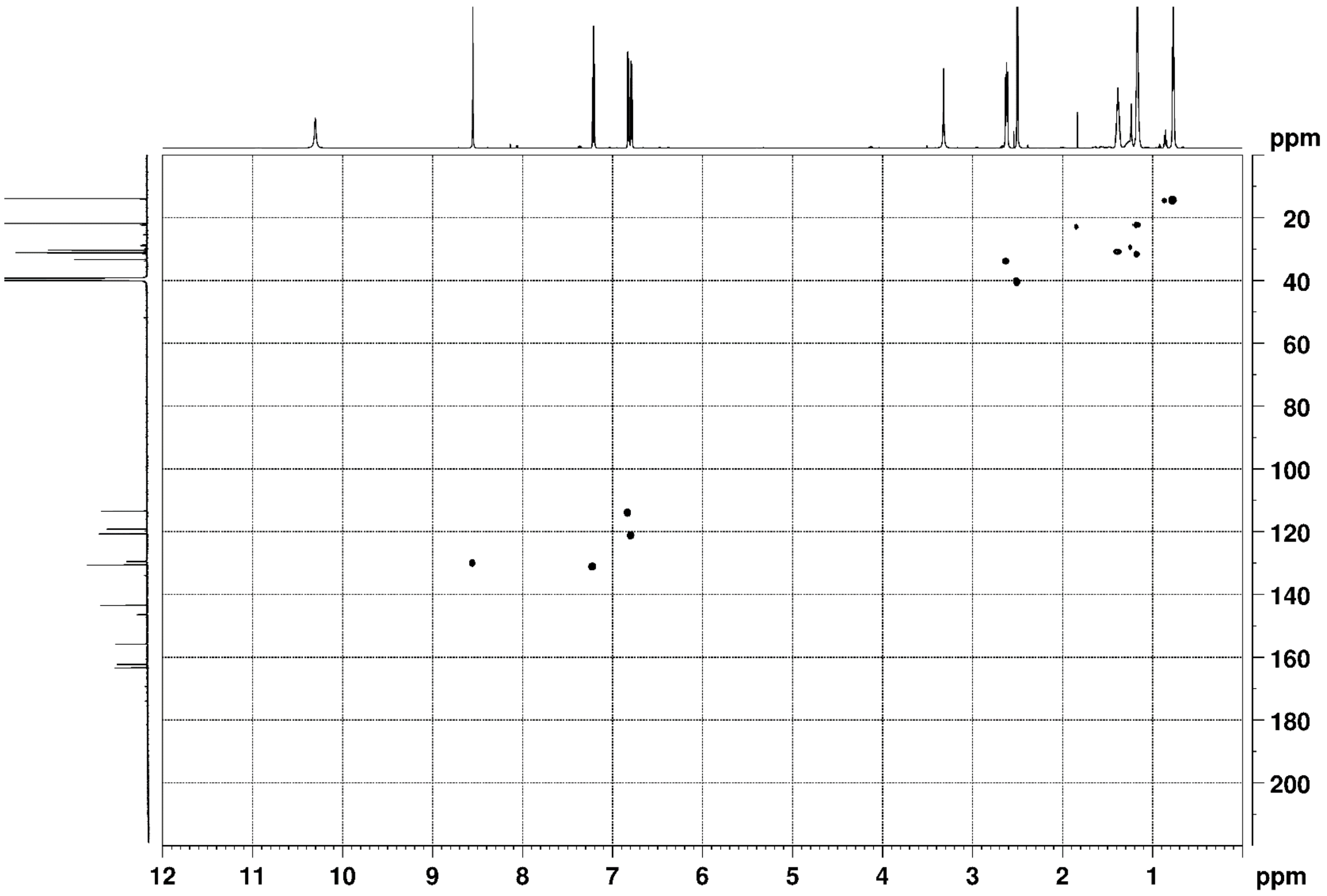

Figure S17: HSQC spectrum of **3** (600 MHz, DMSO-d_6, 25 °C)

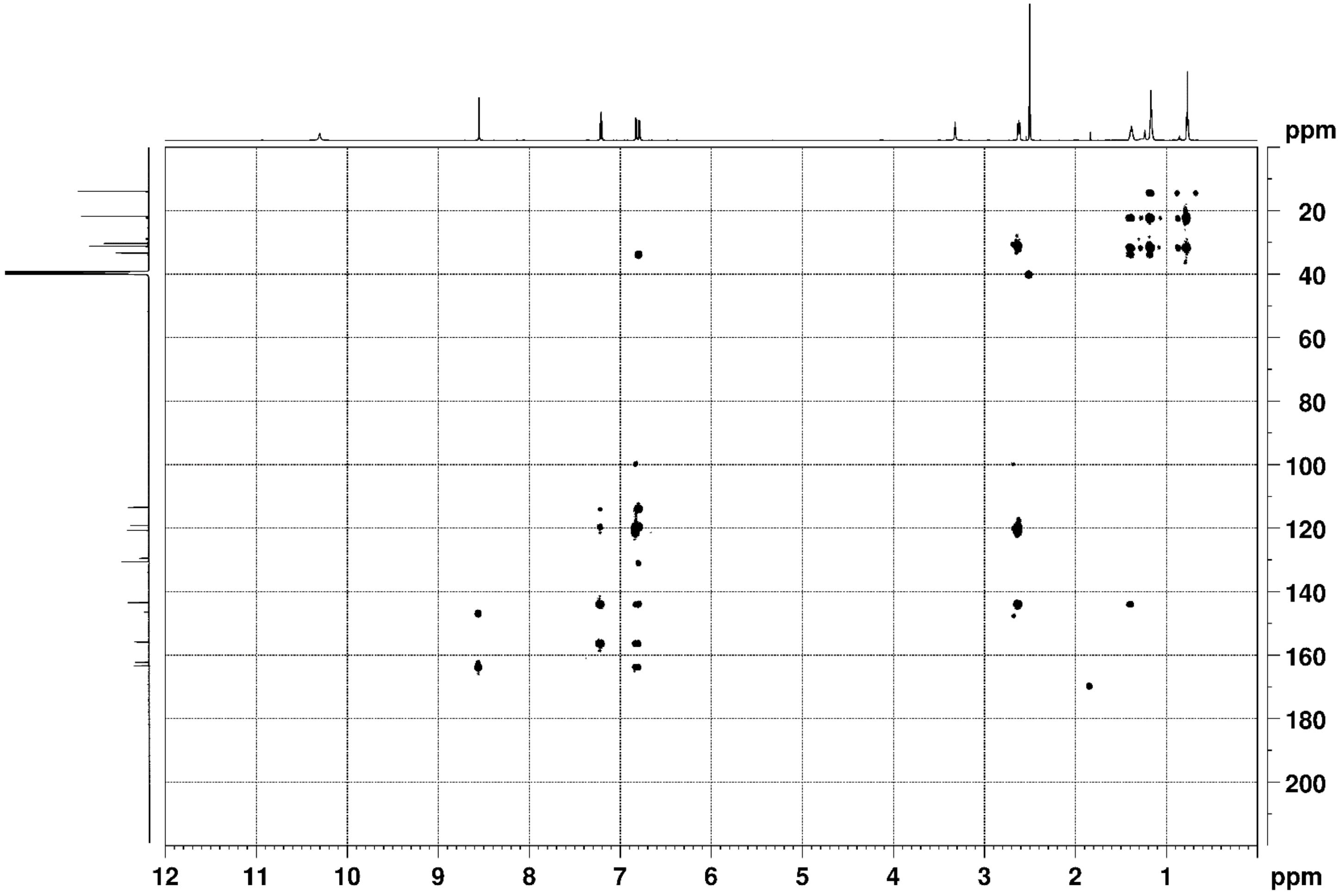

Figure S18: HMBC spectrum of **3** (600 MHz, DMSO-d_6, 25 °C)

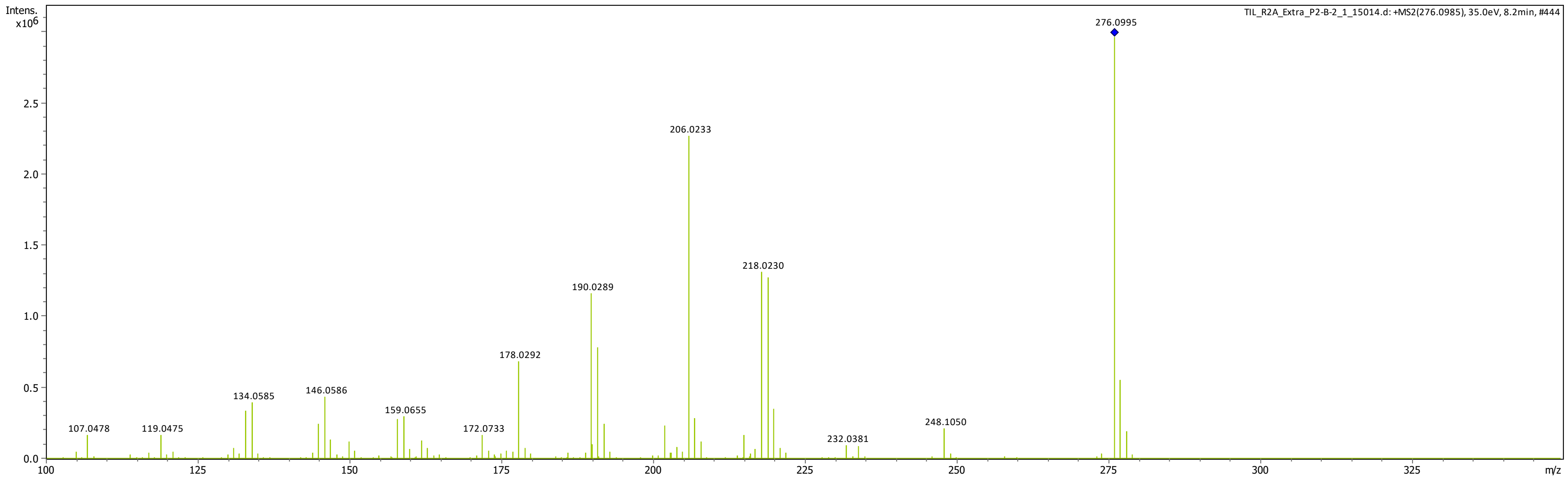

Figure S19: MS/MS spectrum of **4**

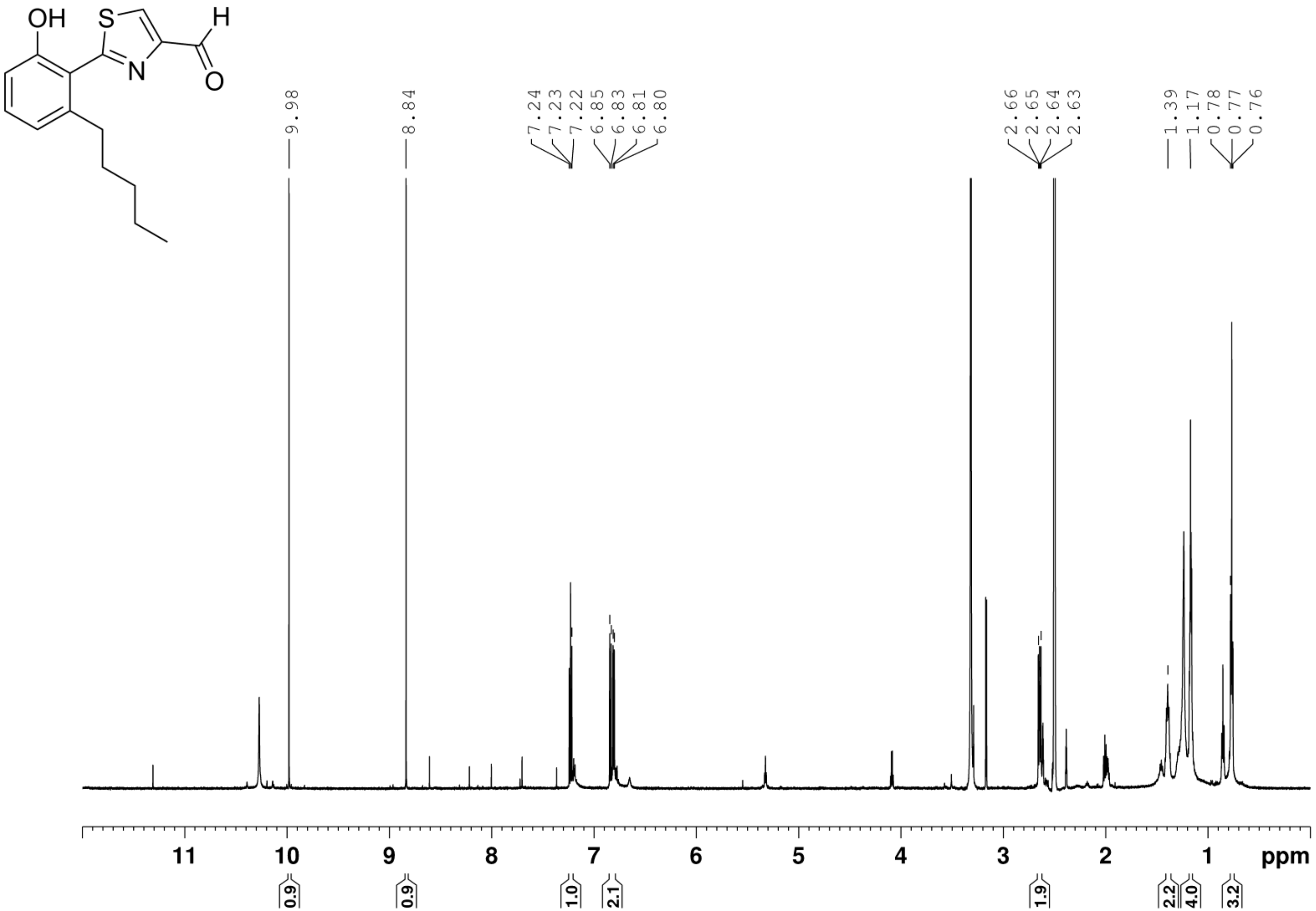

Figure S20: ^{1}H NMR spectrum of **4** (600 MHz, DMSO-d_6, 25 °C)

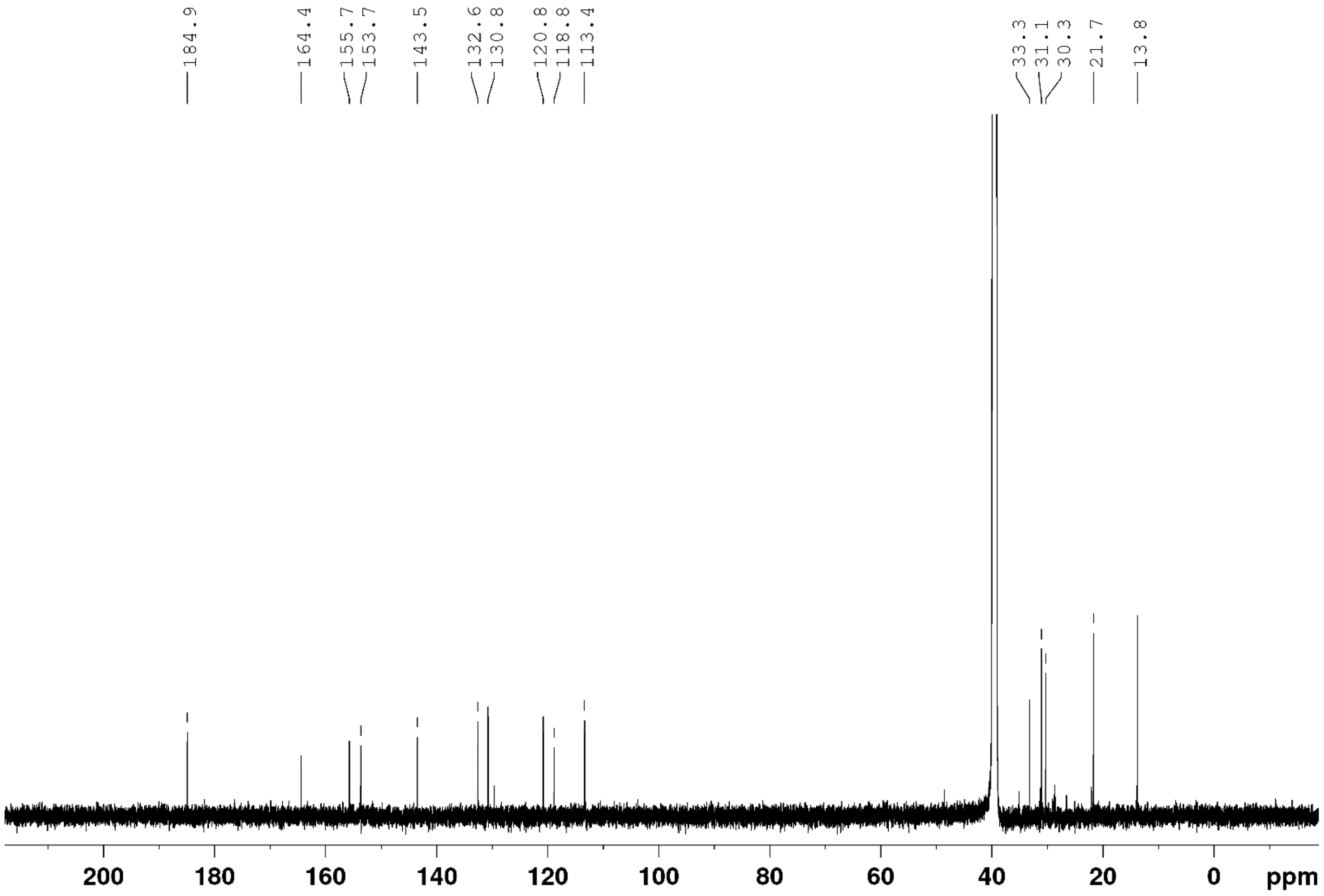

Figure S21: ^{13}C NMR spectrum of **4** (150 MHz, DMSO-d_6, 25 °C)

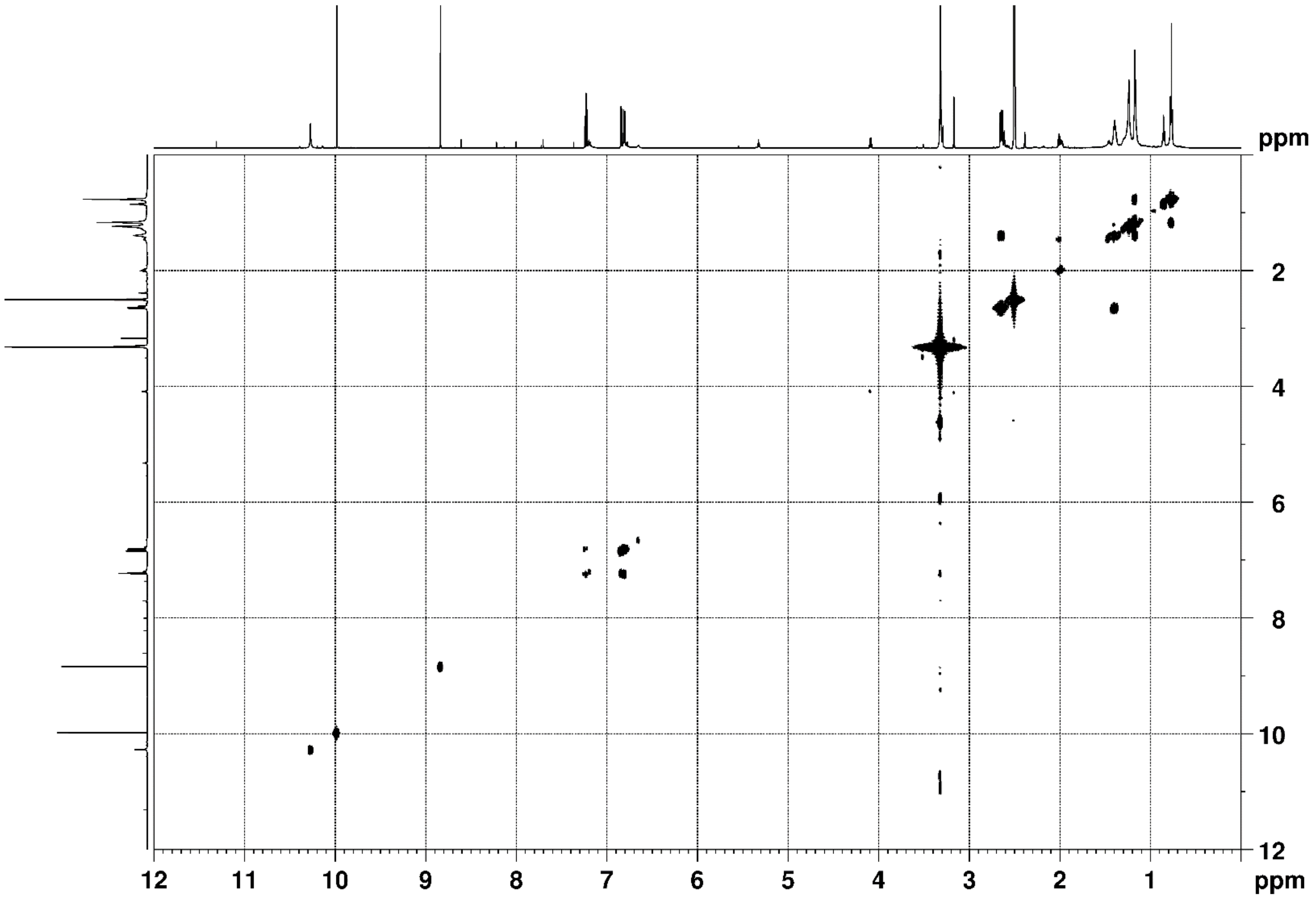

Figure S22: COSY spectrum of **4** (600 MHz, DMSO-d_6, 25 °C)

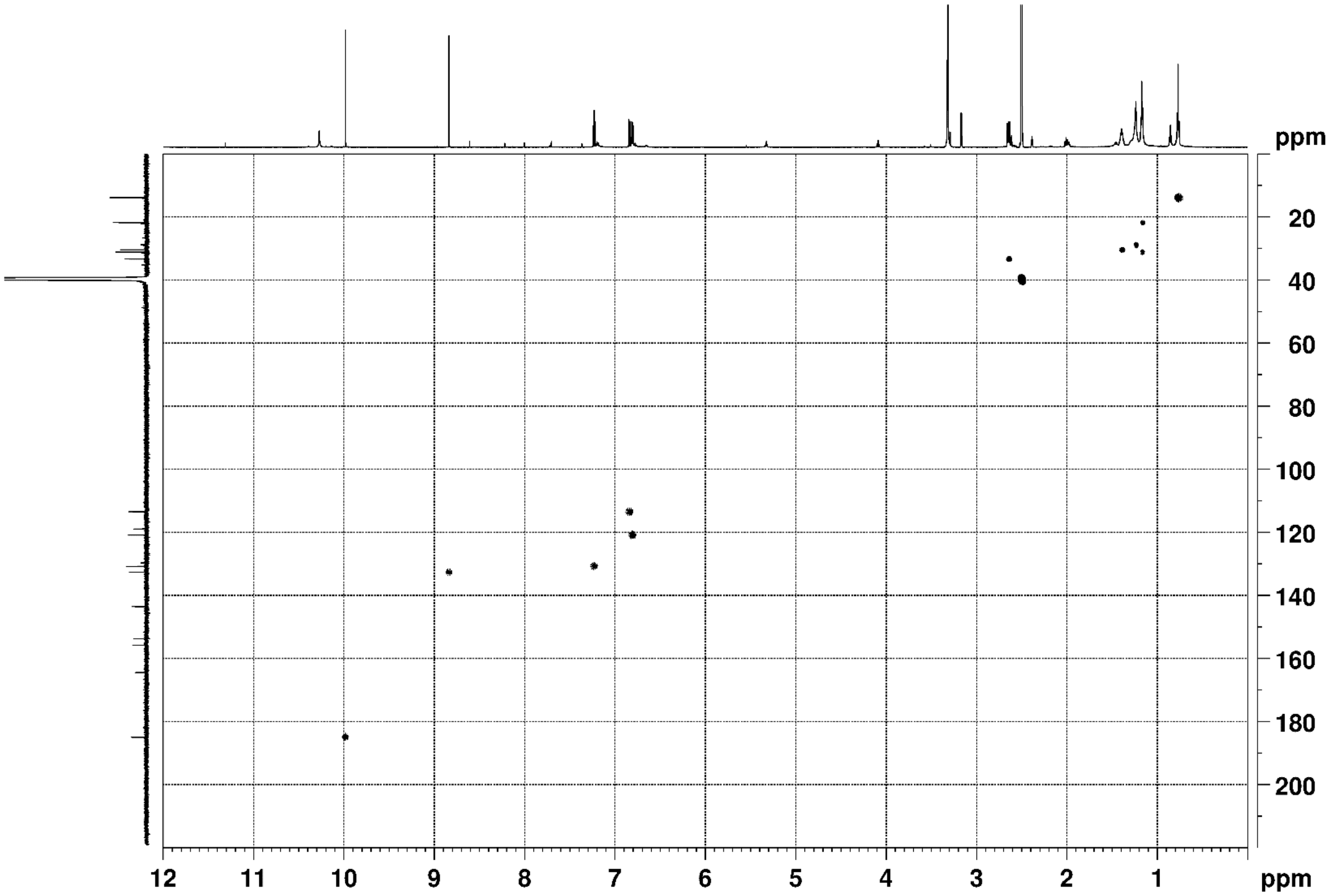

Figure S23: HSQC spectrum of **4** (600 MHz, DMSO-d_6, 25 °C)

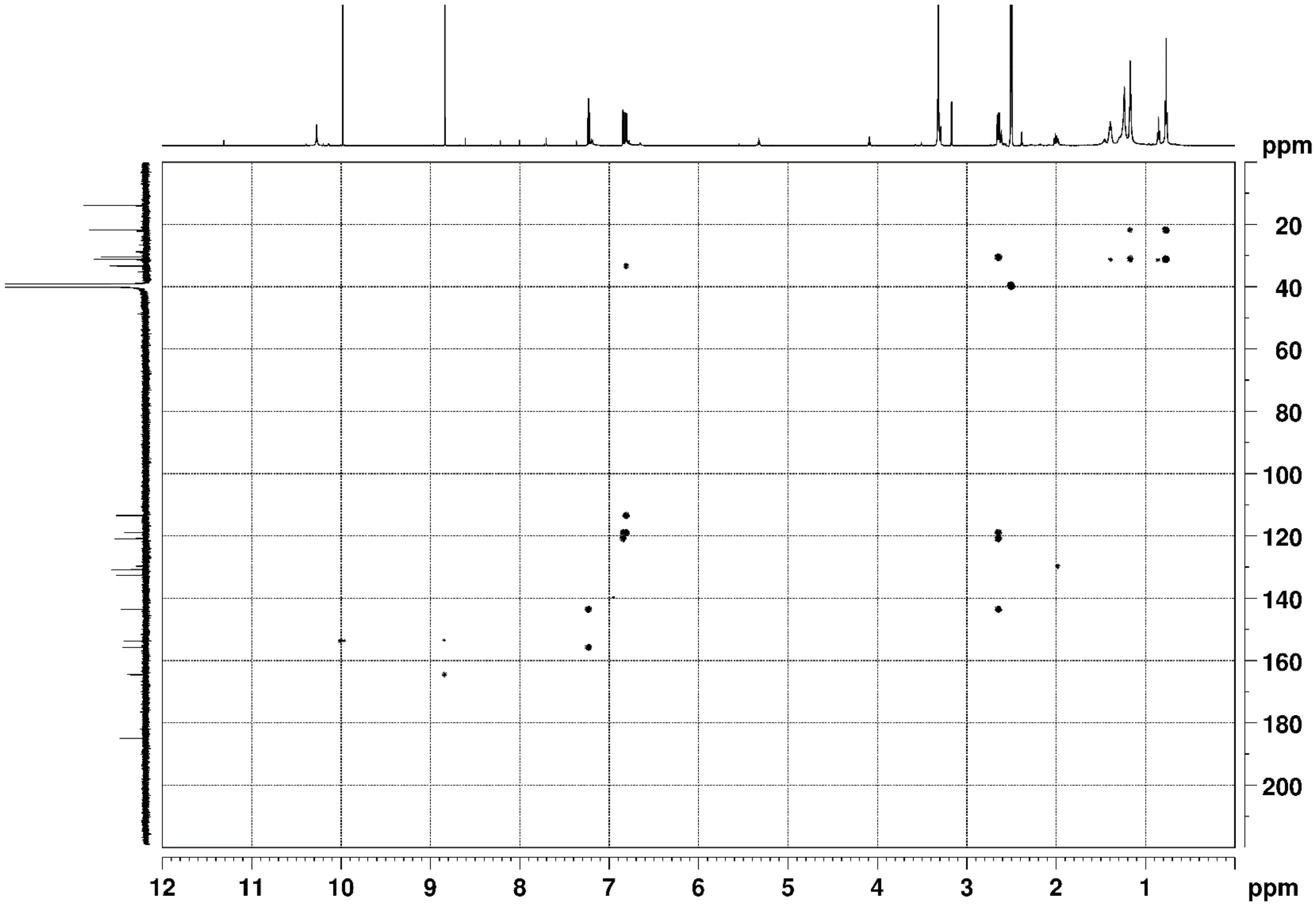

Figure S24: HMBC spectrum of **4** (600 MHz, DMSO-d_6, 25 °C)

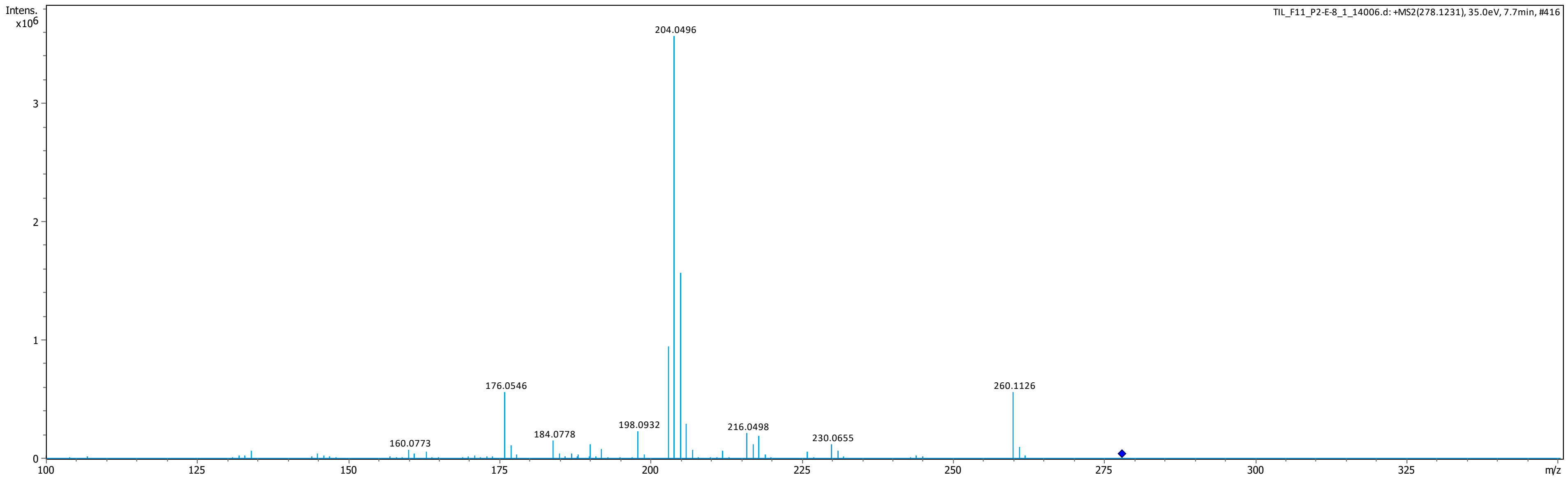

Figure S25: MS/MS spectrum of **5**

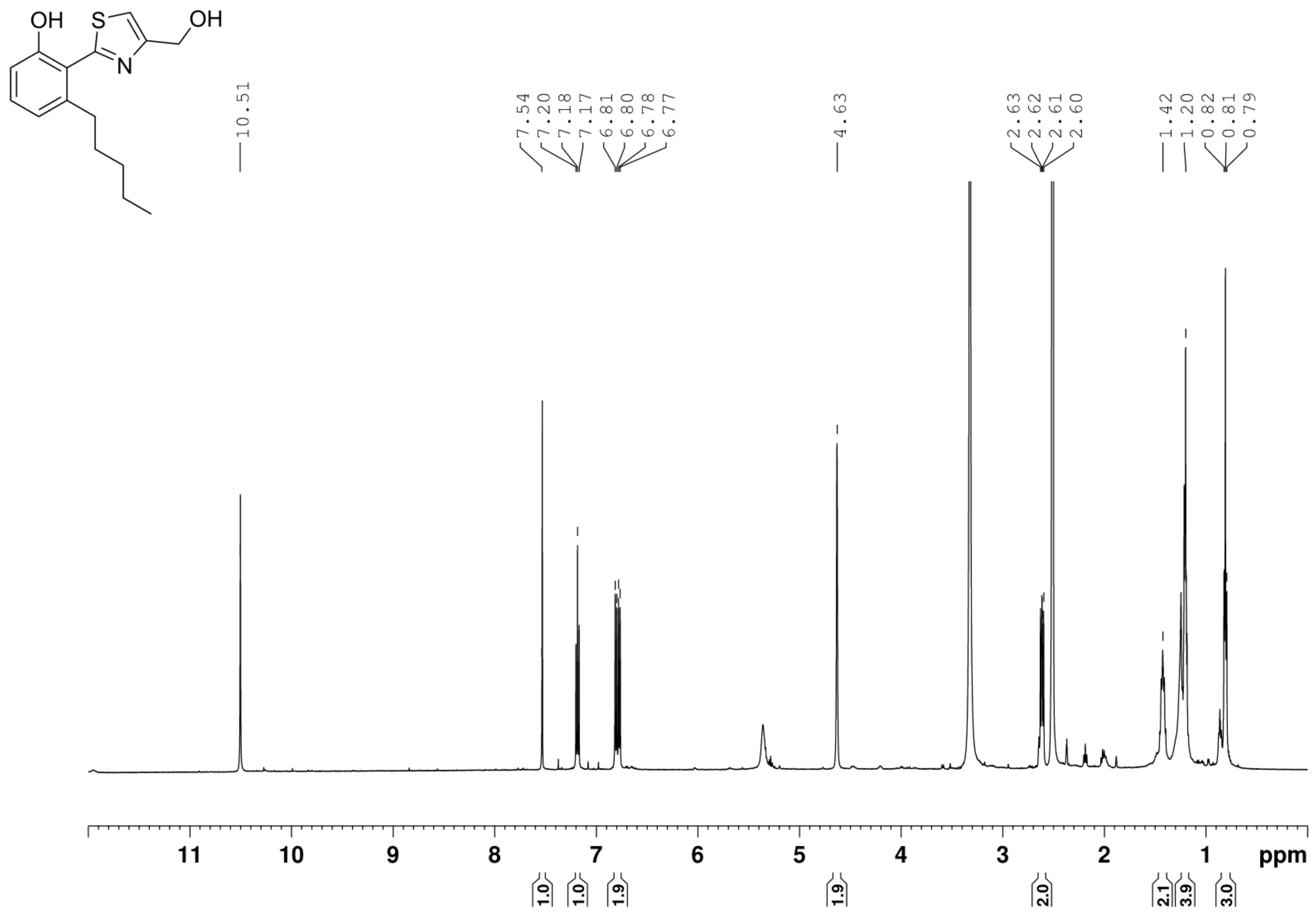

Figure S26: ^{1}H NMR spectrum of **5** (600 MHz, DMSO-d_6, 25 °C)

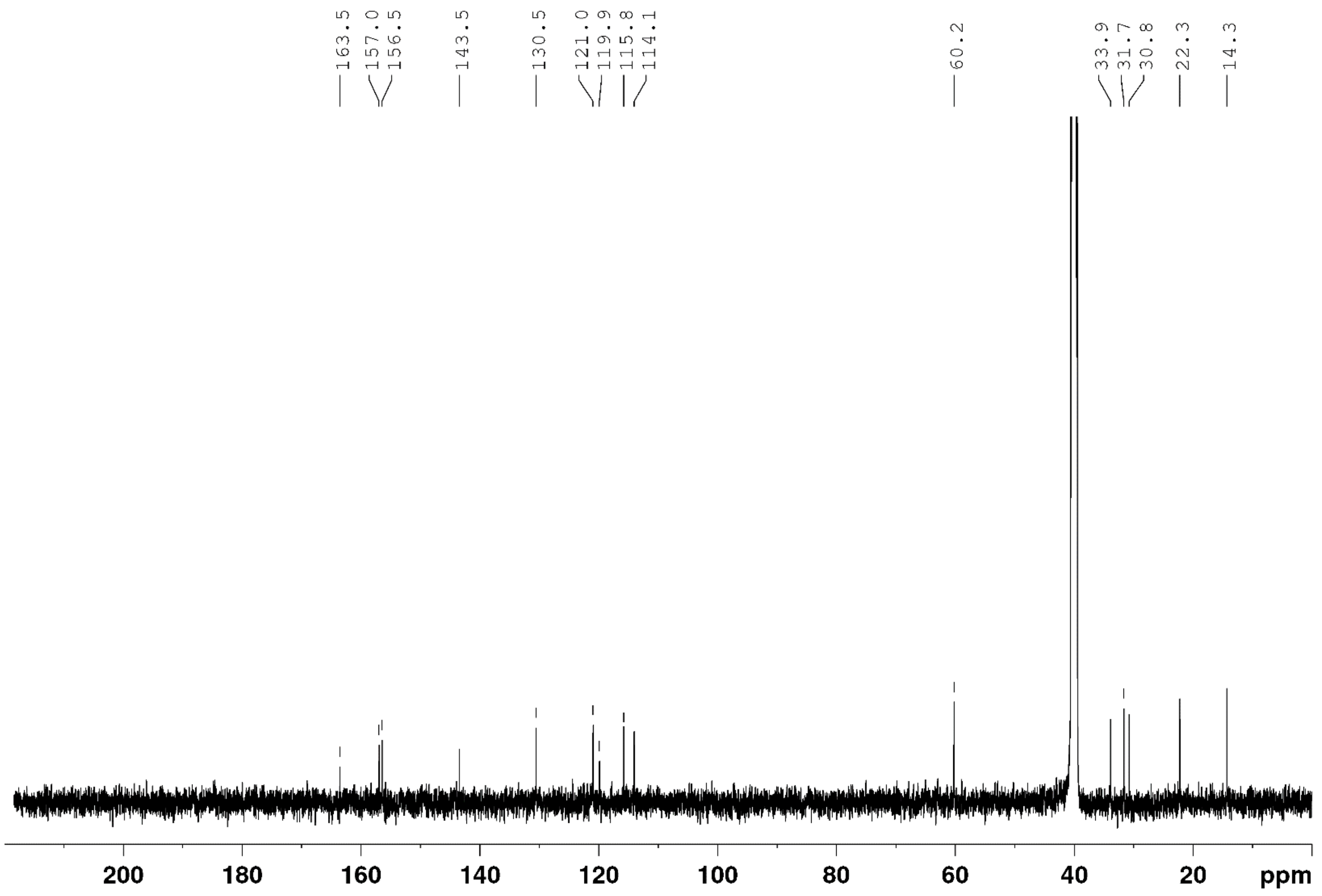

Figure S27: ^{13}C NMR spectrum of **5** (150 MHz, DMSO-d_6, 25 °C)

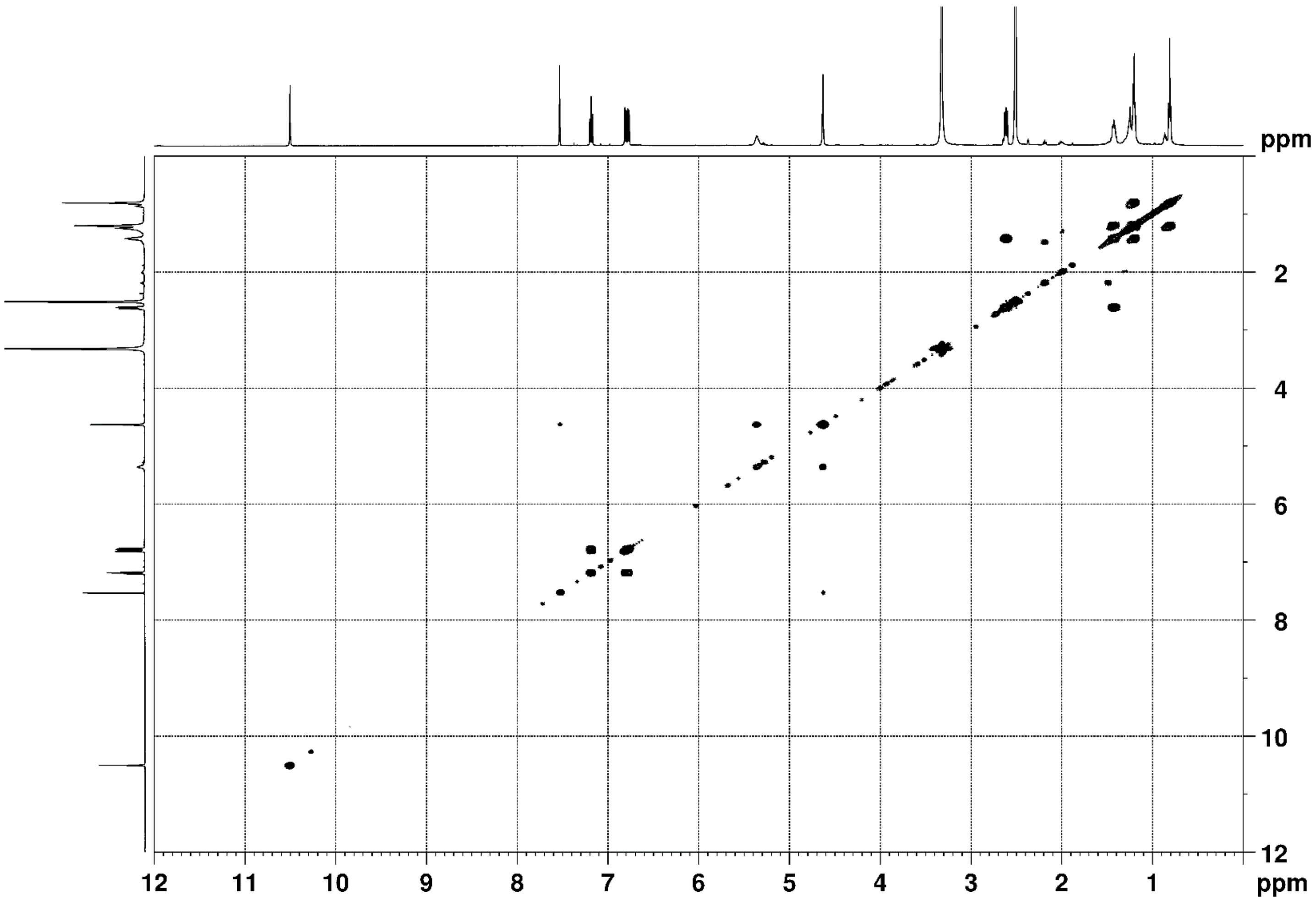

Figure S28: COSY spectrum of **5** (600 MHz, DMSO-d_6, 25 °C)

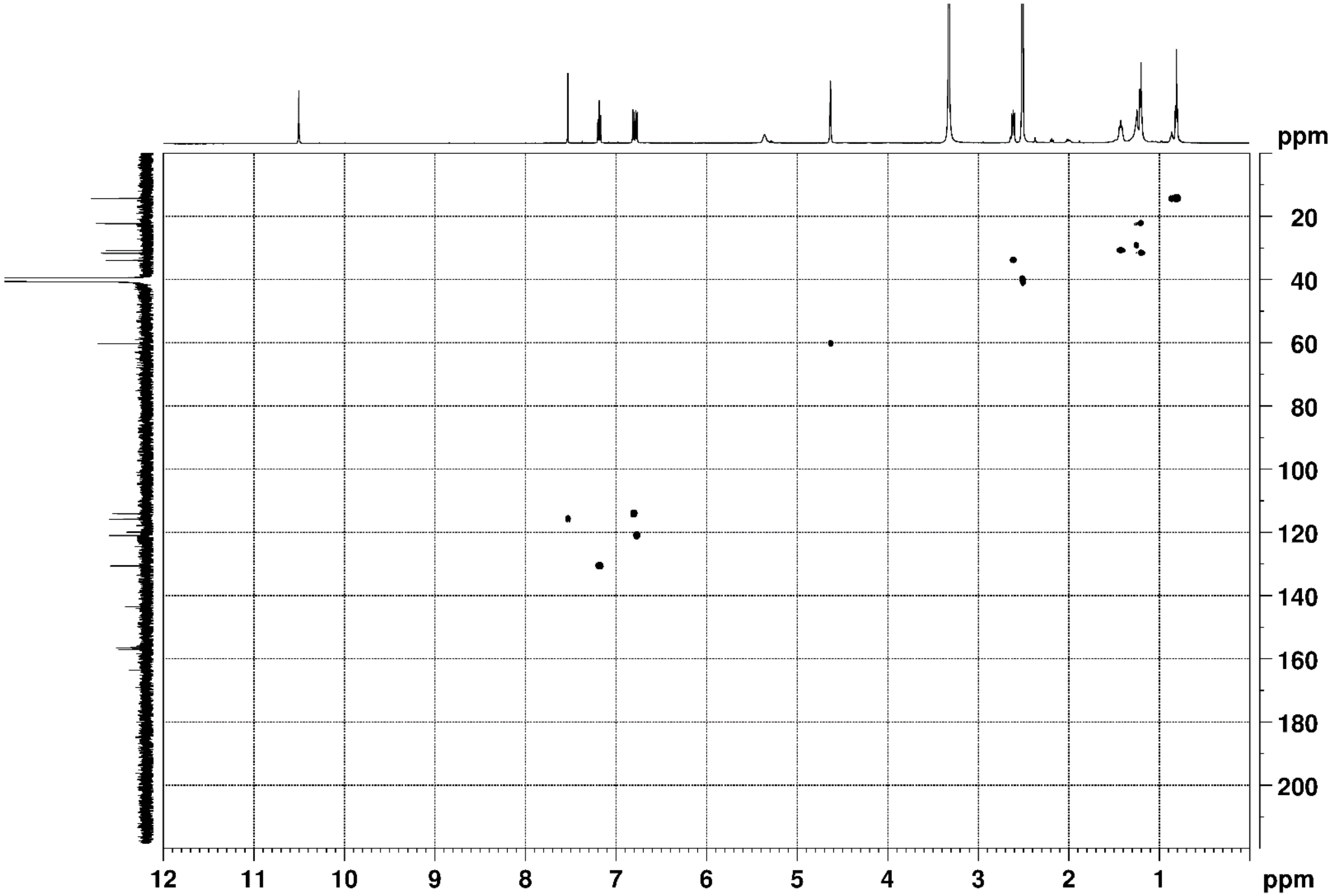

Figure S29: HSQC spectrum of **5** (600 MHz, DMSO-d_6, 25 °C)

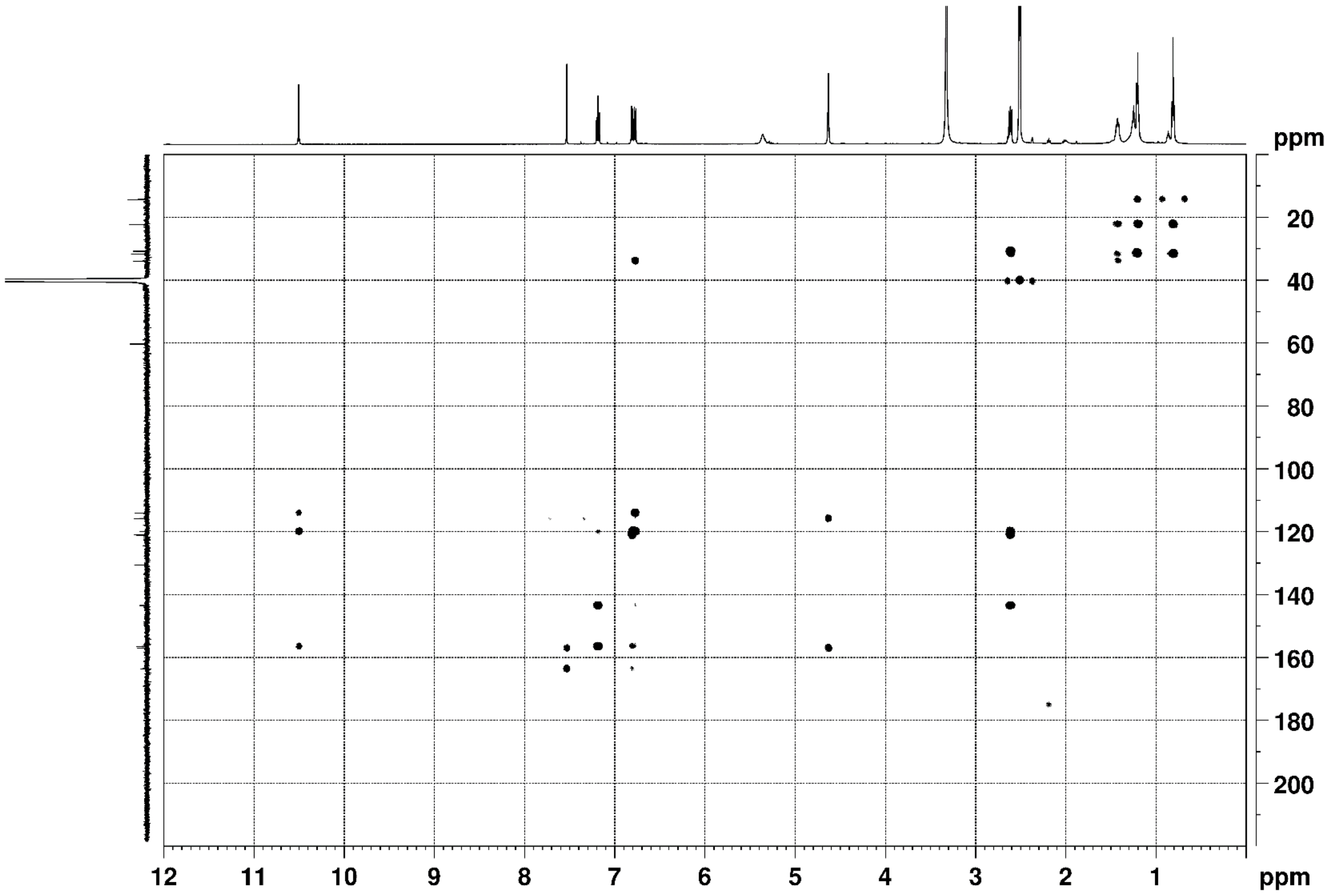

Figure S30: HMBC spectrum of **5** (600 MHz, DMSO-d_6, 25 °C)

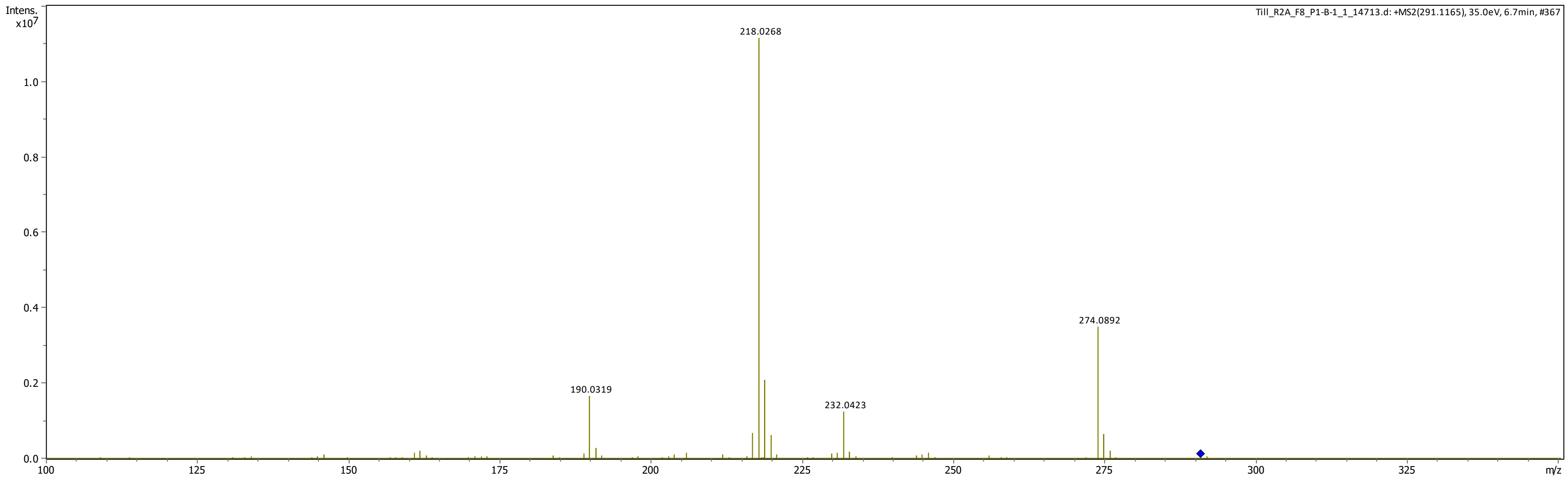

Figure S31: MS/MS spectrum of **6**

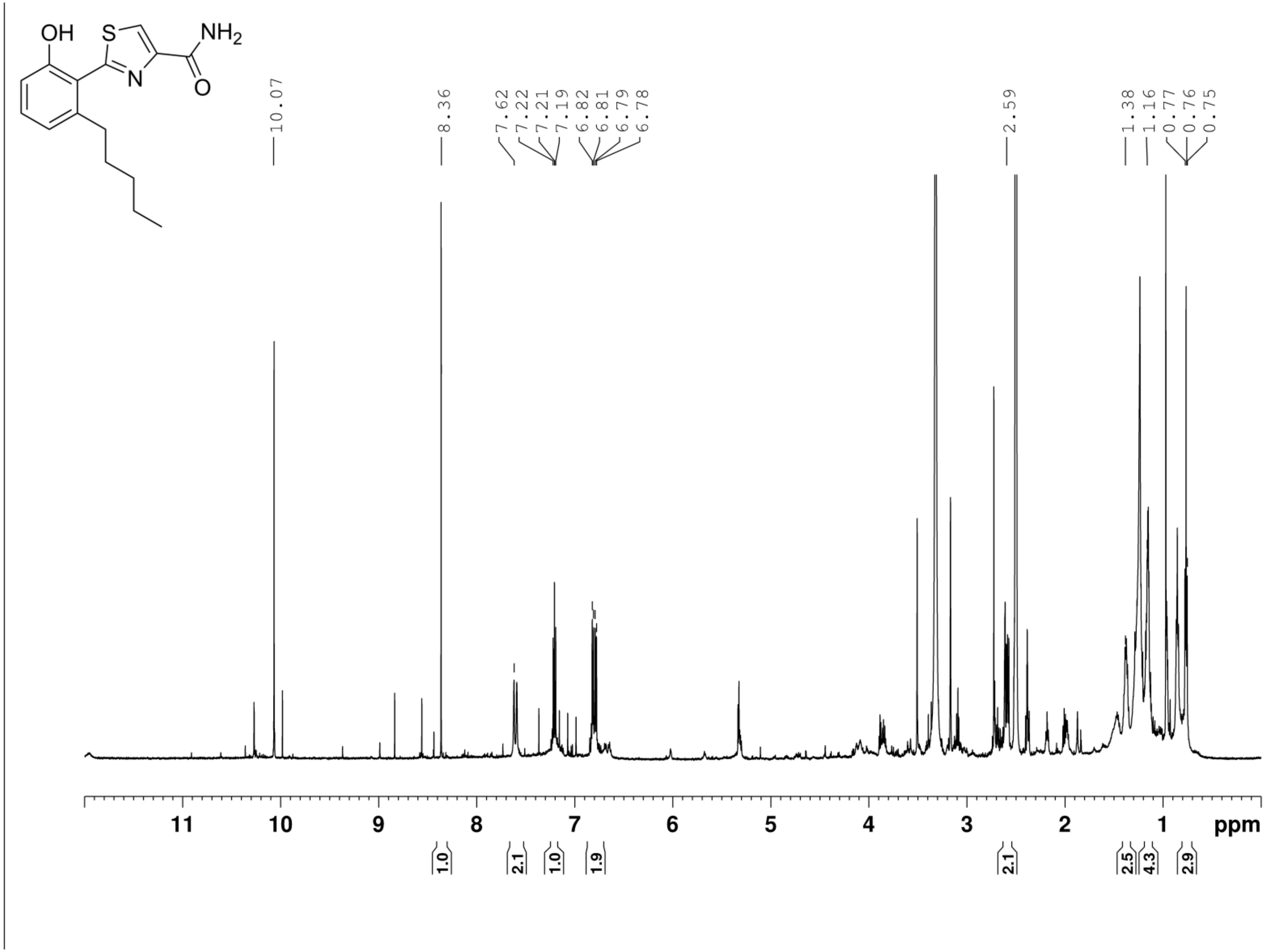

Figure S32: ^{1}H NMR spectrum of **6** (600 MHz, DMSO-d_6, 25 °C)

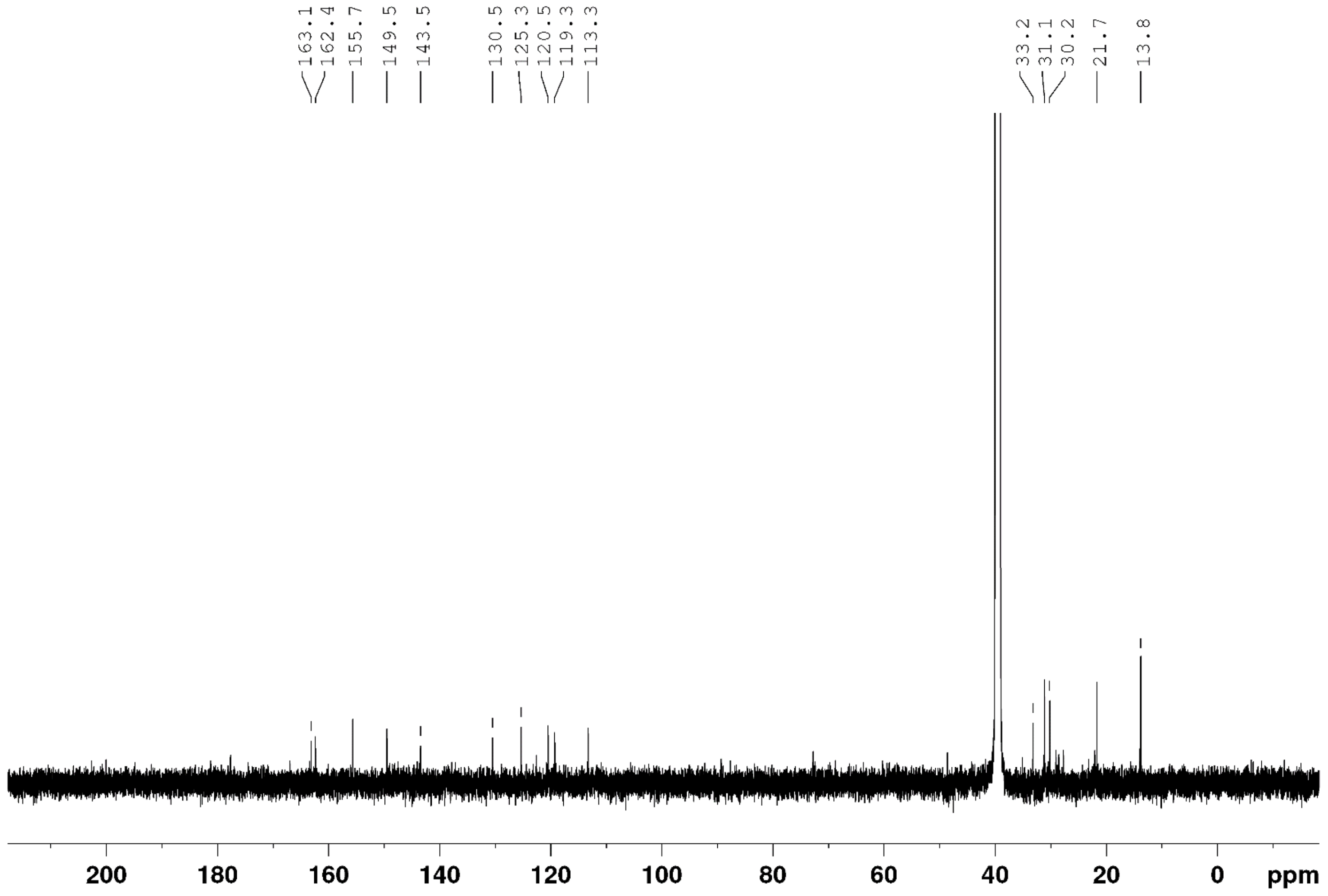

Figure S33: ^{13}C NMR spectrum of **6** (150 MHz, DMSO-*d*$_6$, 25 °C)

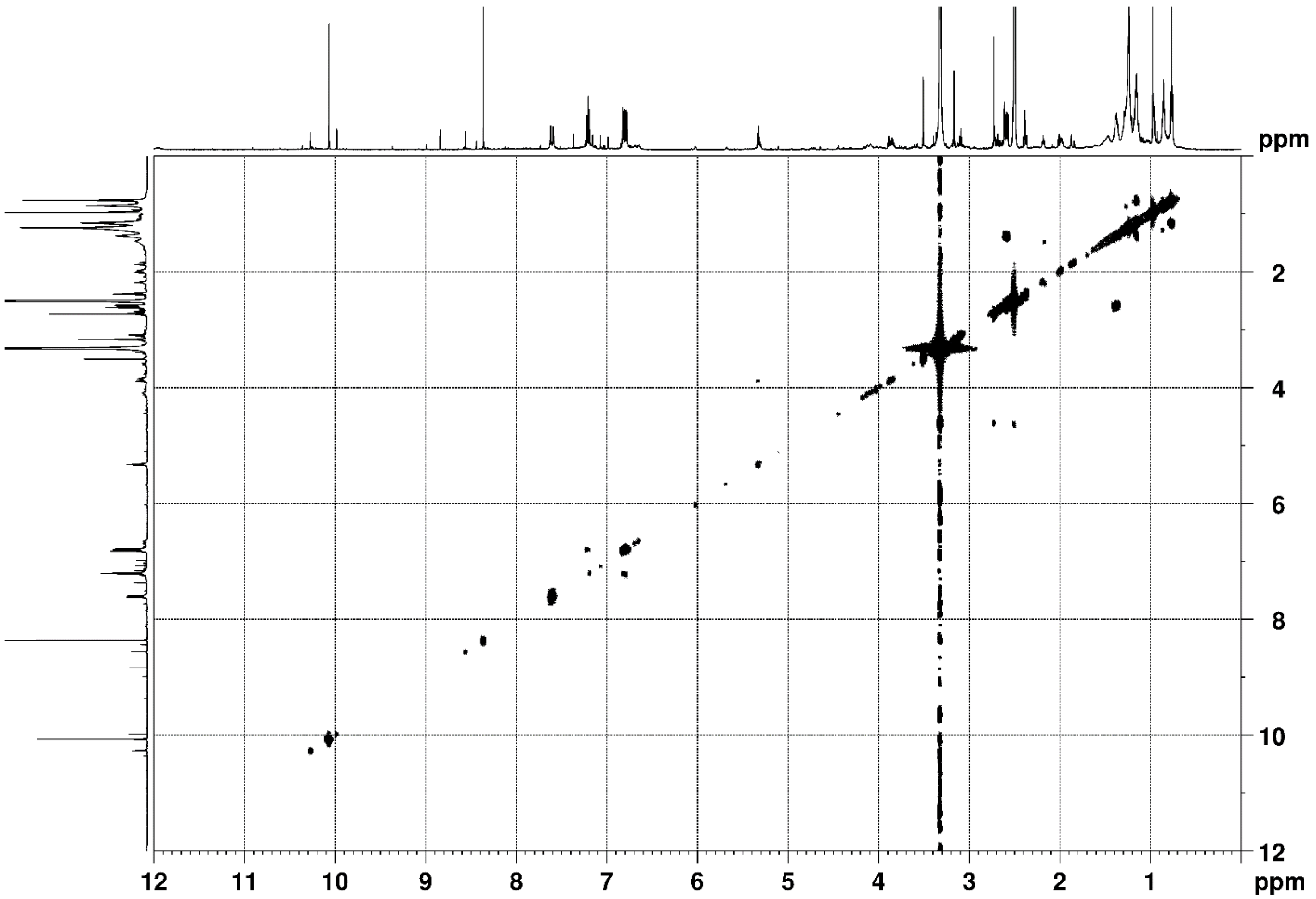

Figure S34: COSY spectrum of **6** (600 MHz, DMSO-d_6, 25 °C)

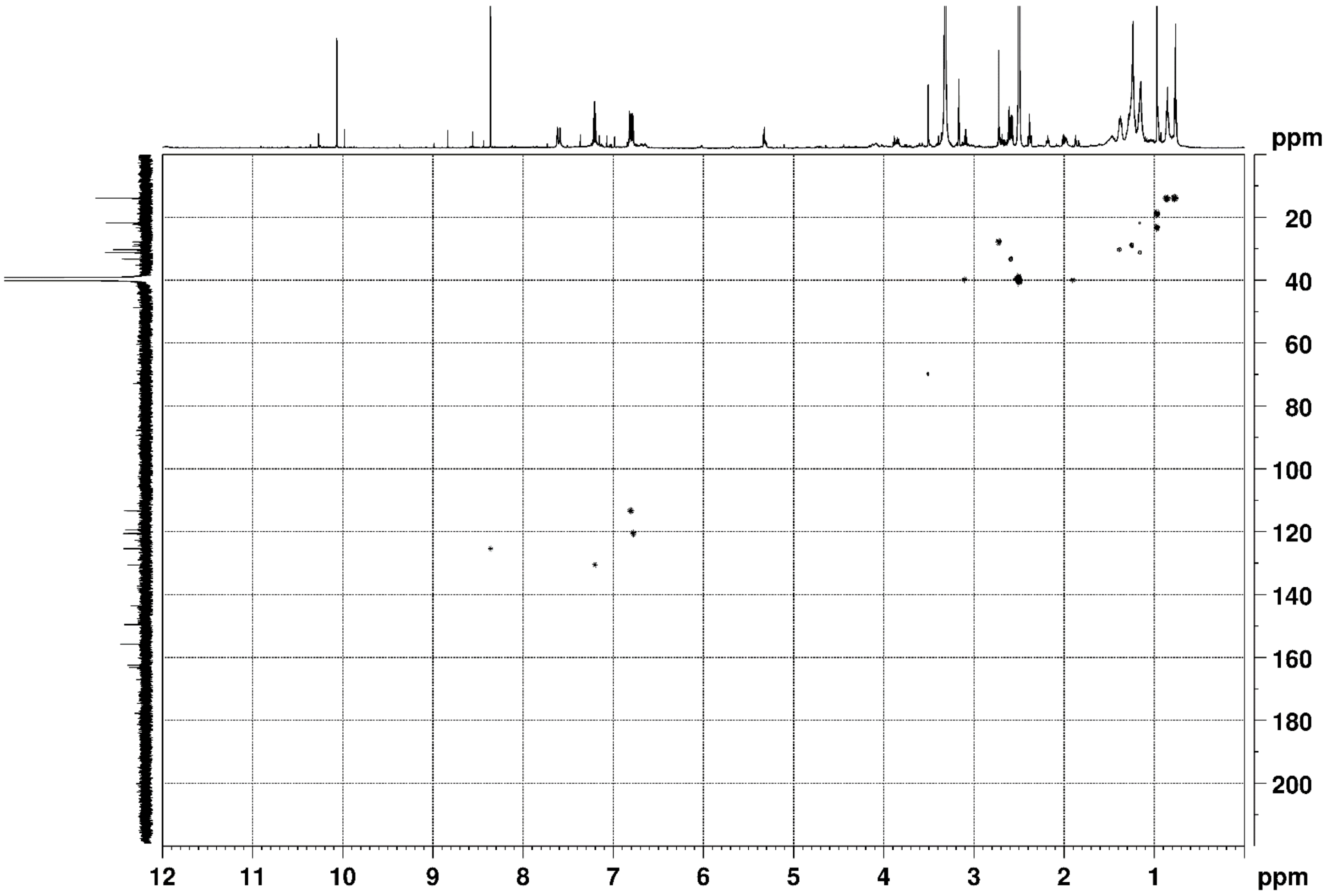

Figure S35: HSQC spectrum of **6** (600 MHz, DMSO-d_6, 25 °C)

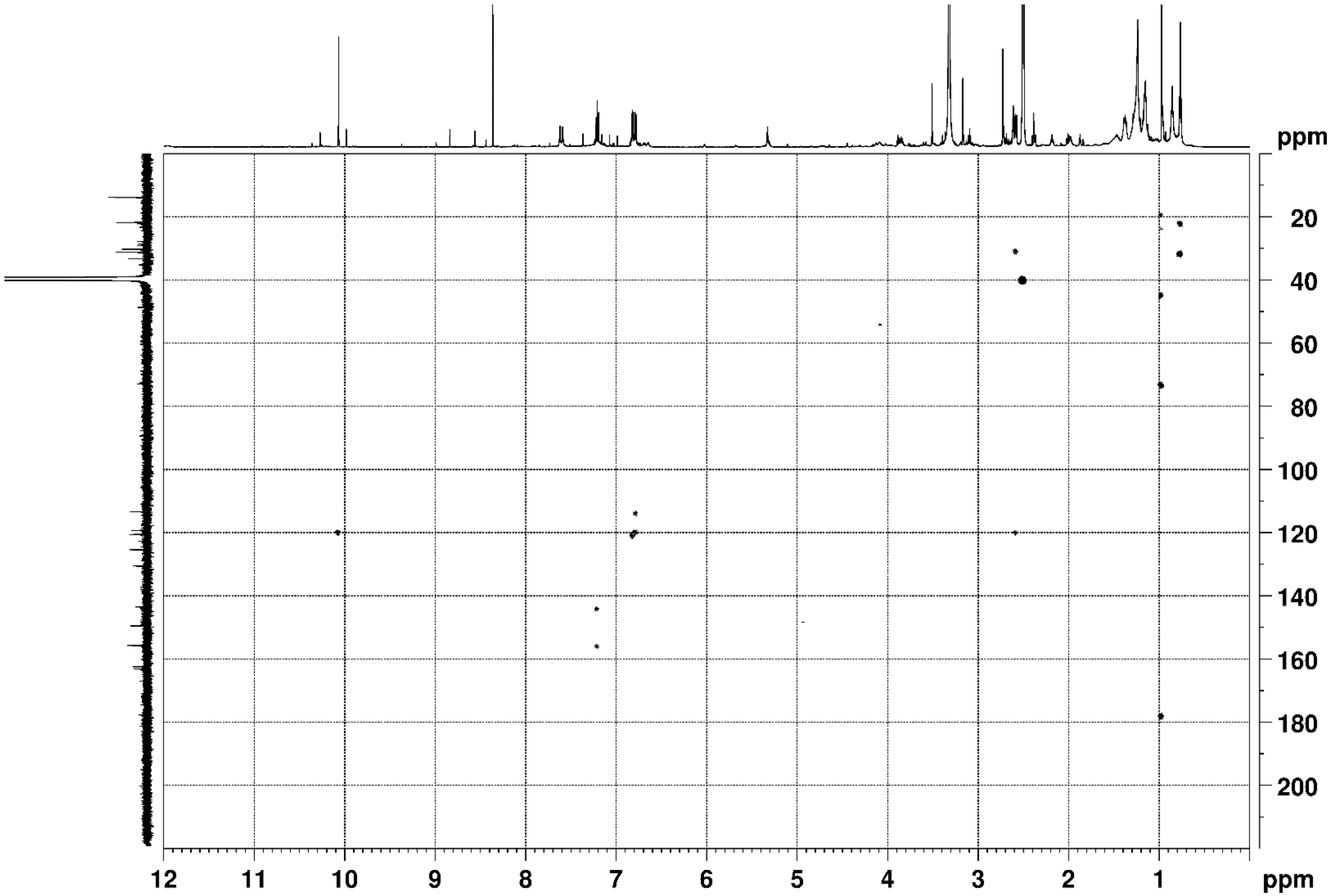

Figure S36: HMBC spectrum of **6** (600 MHz, DMSO-d_6, 25 °C)
